# Shear behavior of UHPFRC deep beams with internal opening reinforcement

**DOI:** 10.1038/s41598-026-62381-x

**Published:** 2026-07-19

**Authors:** Ahmed M. Yousef, Ahmed M. Tahwia, Ali A. Elzain

**Affiliations:** Structural Engineering Department, Faculty of Engineering, Mansoura University, Mansoura, 35516 Egypt

**Keywords:** Shear behavior, UHPFRC, Deep beams, Opening reinforcement, Failure modes, Numerical modeling, Engineering, Materials science

## Abstract

This study experimentally and numerically investigated the shear behavior of UHPFRC deep beams with internal-opening reinforcement, focusing on how internal-opening reinforcement detailing can mitigate opening-induced shear capacity loss. The test program included eight simply supported UHPFRC deep beams tested under four-point loading and arranged into two groups with different geometries. Group I comprised five beams with an *a/d* ratio of 0.61, including one solid reference beam and four beams with a square opening within the shear span, and intersected the load path. Group II comprised three beams with an *a/d* ratio of 0.79 and different opening sizes. Three internal opening reinforcement techniques with different ratios were examined: (1) additional vertical and horizontal bars around the opening (*µ*_*av*_ = *µ*_*ah*_ = 2.7% or 2.4%), (2) additional stirrups (*ρ*_*va*_ = 4.2% or 5.6%) with vertical and horizontal bars around the opening (*µ*_*av*_ = *µ*_*ah*_ = 2.7% or 2.4%), and (3) diagonal cross-bars around the opening (*µ*_*ax*_ = 1.8%). The results showed that the unreinforced opening reduced the cracking load by 41.4% and the ultimate load by 40.4%, compared with the solid beam. Techniques (1), (2), and (3) increased ultimate shear capacity by 16.5%, 69.2%, and 25.3%, respectively, versus the unreinforced opening beam, achieving approximately 54.6%, 79.4%, and 58.8% recovery of the solid-beam capacity. A 3D numerical model using concrete damage plasticity reproduced damage and load–deflection responses, with mean experimental-to-numerical ratios of 1.08 for ultimate shear load and 1.30 for midspan deflection, supporting the model’s predictive reliability for deep beams with internal opening reinforcement.

## Introduction

Ultra-High-Performance Fiber-Reinforced Concrete (UHPFRC) has emerged as a revolutionary cementitious composite material with remarkable mechanical properties and durability, positioning it at the forefront of modern construction technologies. This advanced material is characterized by compressive strengths exceeding 130 MPa, superior tensile capacity, enhanced ductility, and remarkable resistance to environmental degradation^1–3^. The incorporation of steel fibers into the UHPFRC matrix significantly enhances tensile strength, leading to a strain-hardening response and multi-cracking patterns that distinguish it from conventional concrete materials^4–9^. These outstanding properties stem from its optimized mix design, which includes high cement content, low water-to-cement ratio, fine aggregates, pozzolanic materials, and dispersed fiber reinforcement, resulting in a dense microstructure^10–14^. The unique combination of high strength, low permeability, and crack-free performance under service loads has established UHPFRC as an ideal material for rehabilitation and strengthening applications, particularly in critical infrastructure elements such as bridge deck slabs, deep beams, columns, and structural members subjected to severe mechanical and environmental loads.

Deep beams are an essential class of structural members, widely employed in high-rise buildings, transfer girders, cap beams, bridge decks, pile caps, and shear walls. The capacity of UHPFRC deep beams to transfer heavy loads over short spans makes them indispensable in modern construction, especially when architectural and service constraints necessitate web openings for ducts, pipes, and cables. The introduction of openings within the shear span of shallow or deep beams, however, imposes considerable challenges: openings interrupt the natural load path and diagonal compression strut, reduce shear strength, increase stress concentrations, and impair both the serviceability and safety margins of the structure^15–20^. Mabrouk et al.^15^ examined the structural performance of reinforced concrete deep beams with openings under vertical loads using the strut-and-tie model. Their results indicate that openings significantly disrupt load-transfer mechanisms and adversely affect strength and cracking response, supporting the use of strut-and-tie-based approaches for more reliable design of deep beams with openings. A great number of experimental and numerical investigations have been conducted on the behaviour of solid deep beams constructed from UHPFRC^21–33^. Mirzaaghabeik et al.^24^ highlight that the shear performance of UHPC deep beams is governed not only by material composition but also by geometric proportions of the member. In addition, the results suggest that synthetic fibers can achieve an acceptable structural response compared with steel fibers, supporting their potential use in durable and cost-effective UHPC deep-beam applications.

The Strut-and-Tie Method (STM) is widely recognized as a rational framework for deep beams, where load transfer is governed by compression struts, tension ties, and nodal zones rather than by classical beam theory^34–37^. Its value is greatest in disturbed regions, including beams with web openings, because it provides a clear representation of the internal force path and highlights the regions most vulnerable to cracking and crushing. However, the reliability of STM depends strongly on how the strut geometry, nodal zones, and reinforcement ties are idealized, which makes its predictions sensitive to modeling assumptions. Recent studies have shown that modified STM formulations can improve prediction accuracy for deep beams, yet discrepancies remain when openings interrupt the main compression strut or when reinforcement detailing is not fully captured. Therefore, STM should be viewed as a useful theoretical basis for interpreting load transfer in deep beams with openings, but it should ideally be supported by experimental evidence or nonlinear numerical analysis when the stress field is highly disturbed.

Recently, a small number of experimental investigations studied the behaviour of UHPFRC deep beams with openings^38–41^. Smarzewski et al.^38^ experimentally investigated the behavior of hybrid steel-polypropylene fiber-reinforced high-performance concrete deep beams, with and without openings, focusing on varying reinforcement configurations and fiber contents. The results exhibited that hybrid fibers that work as web reinforcement effectively reduced crack widths and increased the load-carrying capacity. However, the reported 28% improvement indicates a moderate recovery rather than full compensation for the structural penalty caused by the opening. Makki et al.^39^ conducted an experimental study investigating the behavior and shear performance of reactive powder concrete (RPC) deep beams with web openings, externally strengthened with carbon fiber-reinforced polymer (CFRP) strips, using varying schemes. The results demonstrated that CFRP-based external strengthening techniques significantly enhance the structural response of RPC deep beam specimens, with ultimate strength improvements ranging from 11% to 94%. However, the wide range of improvement reflects a strong dependence on the strengthening scheme and the opening vulnerability. Elsayed et al.^40^ executed an experimental investigation on the shear behavior of UHPC beams with and without web openings. The study concluded that increasing the steel fiber volume significantly enhanced the shear strength, stiffness, toughness, ductility, and strain of steel reinforcements. This finding highlights the important role of fibers in UHPC. Al-Enezi et al.^41^ conducted an experimental study into the shear capacity of UHPFRC deep beams with web openings. This study reached a similar conclusion for UHPFRC deep beams, reporting strength reductions of up to 43% depending on opening geometry and position. This confirms that the severity of the opening effect is governed not only by its presence but also by its size and location relative to the strut mechanism. Taken together, these studies indicate that although material enhancement and external strengthening can mitigate damage, the structural response remains highly sensitive to opening configuration, and a more efficient internal reinforcement strategy around the opening is still needed.

Various rehabilitation methods have been investigated to restore or enhance the load-carrying capacity and ensure structural stability of deep beams with openings. These methods include externally bonded Fiber-Reinforced Polymer (FRP) sheets, Near-Surface Mounted (NSM) reinforcement, steel plate bonding, Engineered Cementitious Composites (ECC), and Textile-Reinforced Concrete (TRC). Recent investigations by Abadel et al.^42^ showed that externally applied WWM and CFRP strips can substantially enhance the ultimate load of UHPFRC deep beams with square openings, with improvements of up to 63.8% over unstrengthened specimens. While this confirms the value of external retrofitting, internal strengthening is more practical.

Yanga et al.^43^ investigated the effectiveness of various web reinforcement configurations on the structural behavior of normal-strength reinforced concrete continuous deep beams with openings. This study applied several internal-strengthening techniques, including additional straight bars around openings and additional straight and inclined web reinforcement. These techniques help control the width of diagonal cracks and increase load capacity. Fawzy et al.^44^ reached a similar conclusion by showing that diagonal reinforcement around openings has a pronounced effect under cyclic loading, where crack propagation and stiffness degradation are especially critical. In my view, these studies collectively indicate that the most effective strengthening strategies are those that directly intercept the disturbed stress field around the opening, rather than adding external strengthening.

In this study, the influence of various internal additional reinforcement techniques around the web openings in the shear span of UHPFRC deep beams was experimentally and numerically investigated. The main studied parameters were the quantity and shape of additional reinforcement around openings, the opening sizes and locations, and the shear span-to-depth ratio (*a/d*). A 3D numerical model was proposed to simulate the shear behavior of UHPFRC deep beams with different shapes of opening reinforcement.

## Test program

### Tested UHPFRC beams

**Fig. 1:**
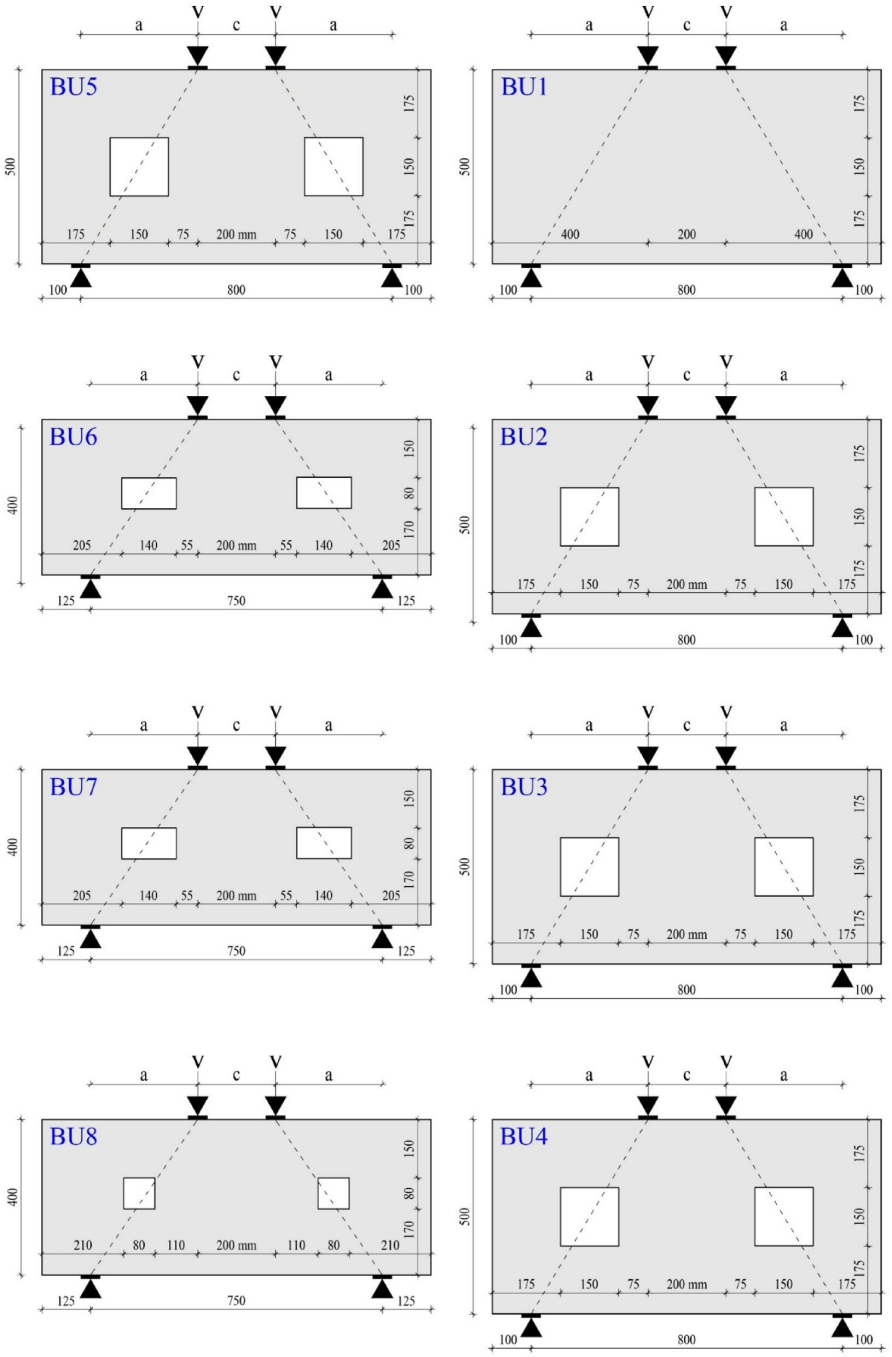
Deep Beams dimensions, openings size, and locations.

**Fig. 2:**
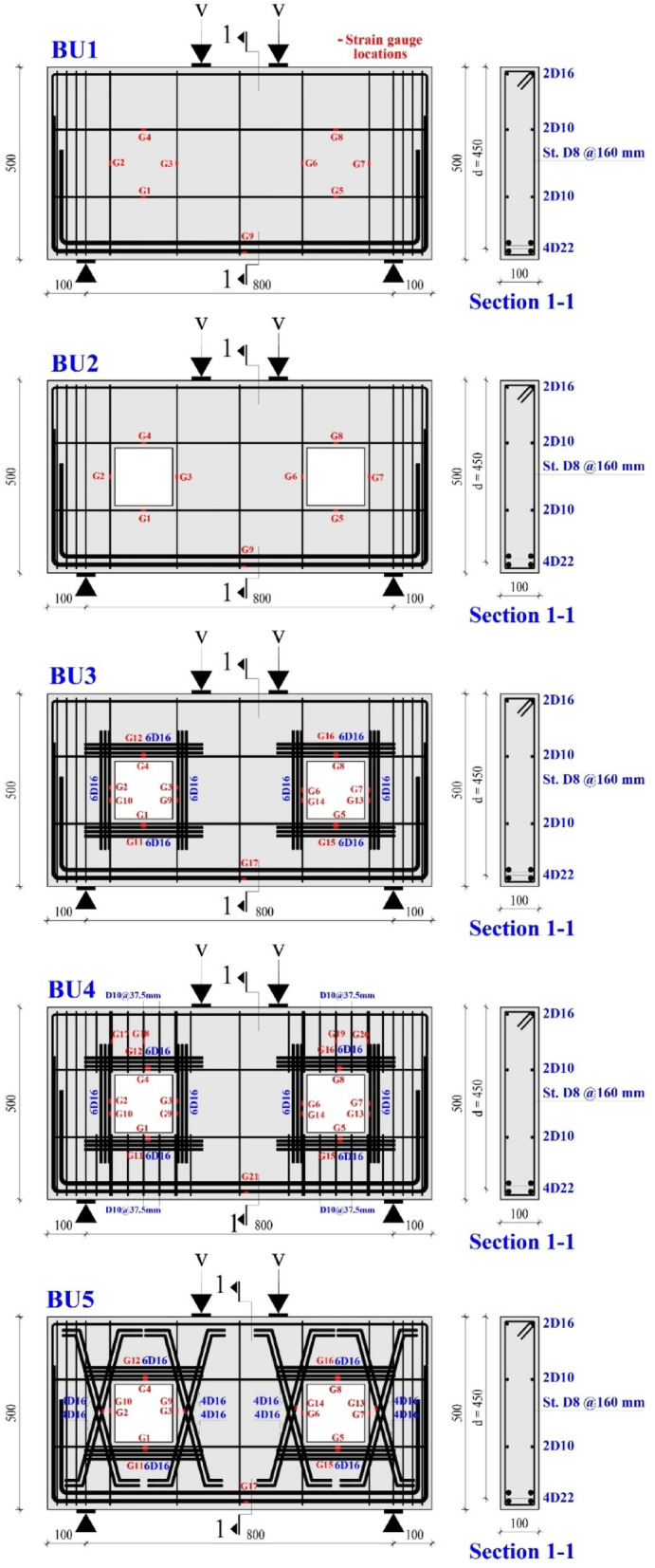
Details of reinforcement of Group I.

**Fig. 3:**
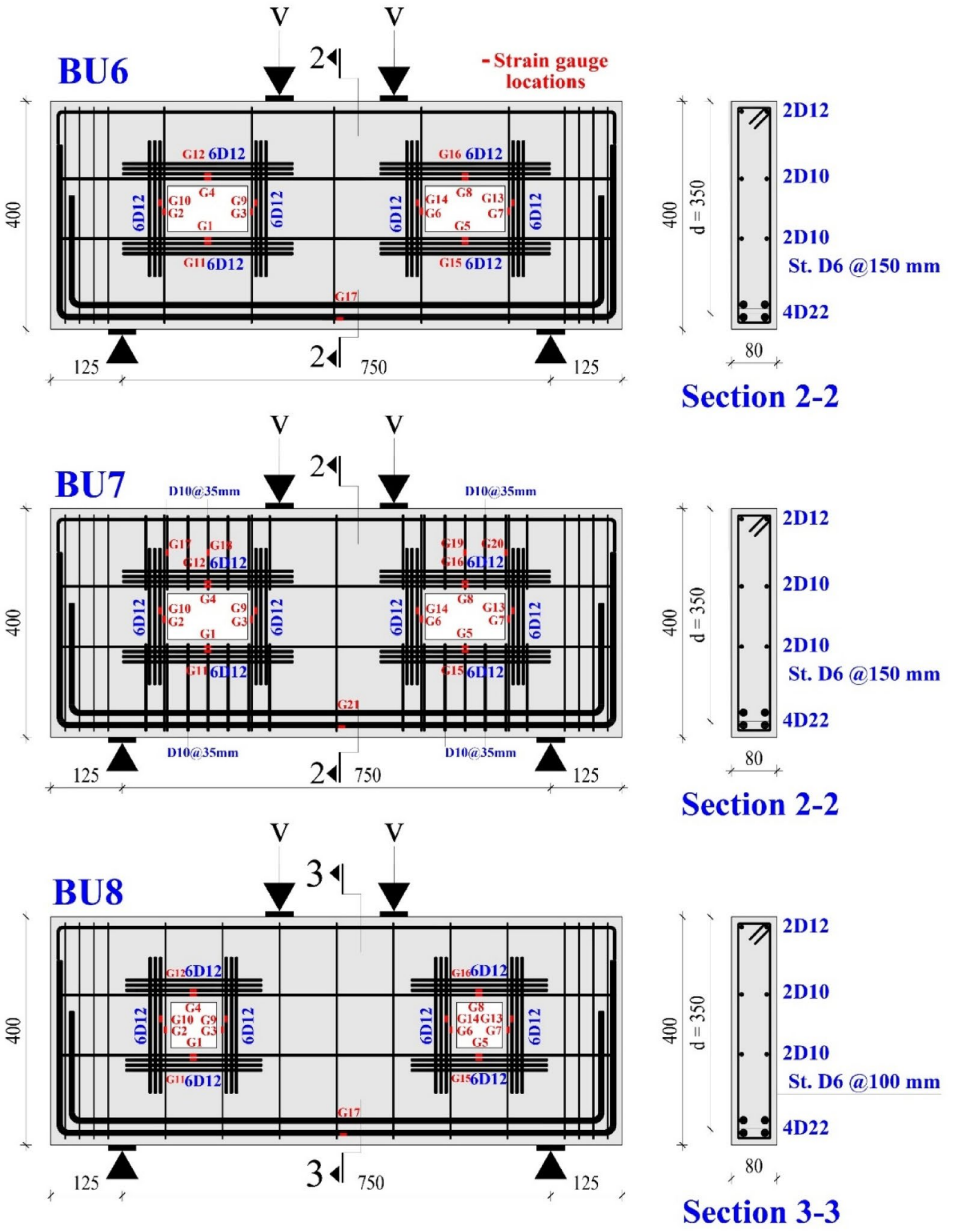
Details of reinforcement of Group II.

The primary objective of this study was to determine the impact of distinct additional reinforcement techniques around openings on UHPFRC deep beams tested in shear. Eight simply supported UHPFRC deep beams were investigated in this research under a four-point bending test. The specimens were symbolized (BU), where B denotes deep beams and U denotes UHPFRC. Based on the investigated international codes (ACI 318, EC-2, and ECP 203)^45–47^, the analyzed beams are listed as deep beams. Figure 1 shows the beam’s layout and dimensions. The experimental program consists of two groups; group I comprises five beams with a length of 1000 mm, a rectangular cross-section of 100 mm × 500 mm, and a clear span of 800 mm. One of them had no opening (a solid beam as a reference), and four had the same opening of 150 mm × 150 mm. All tested deep beams of this group have the same internal longitudinal and web reinforcement but differ in the quantities and shapes of the opening reinforcement, as shown in Fig. 2; Table 1. All the tested five beams had a shear span-to-depth ratio (*a/d*) of 0.61. Group II consists of three beams with a length of 1000 mm, a rectangular cross-section of 80 mm × 400 mm, and a clear span of 750 mm, with different opening sizes and additional reinforcement techniques. All beams in group II had a shear span-to-depth ratio (*a/d*) equal to 0.79. Details of the reinforcement of beams of group II are shown in Fig. 3; Table 1.

**Table 1:** Tested beams details.

Group	Beam	*f*_*cu*_ MPa	*f*_*c*_^*‘*^ MPa	Opening size Width × height (mm×mm)	*a/d* ratio	Longitudinal bars		Vertical web RFT			Additional opening RFT		
						Lower	Upper	*s*_*v*_	*d*_*v*_	*ρ*_*v*_ *(%)*	Straight	Stirrups over and under the opening	Cross bars
Ⅰ	BU1	145.3	133.2	solid	0.61	4D22	2D16	160	8	0.63	–	–	–
	BU2	145.3	133.2	150 × 150	0.61	4D22	2D16	160	8	0.63	–	–	–
	BU3	145.3	133.2	150 × 150	0.61	4D22	2D16	160	8	0.63	6D16 (*µ*_*av*_ = *µ*_*ah*_ = 2.7%) (around opening edges)	–	–
	BU4	145.3	133.2	150 × 150	0.61	4D22	2D16	160	8	0.63	6D16 (*µ*_*av*_ = *µ*_*ah*_ = 2.7%) (around opening edges)	D10@ 37.5 mm (*ρ*_*va*_ = 4.2%)	–
	BU5	145.3	133.2	150 × 150	0.61	4D22	2D16	160	8	0.63	6D16 (*µ*_*ah*_ = 2.7%) (upper and lower edge only)	–	4D16 *(µ*_*ax*_ = 1.8%)
II	BU6	145.3	133.2	140 × 80	0.79	4D22	2D12	150	6	0.47	6D12 (*µ*_*av*_ = *µ*_*ah*_ = 2.4%) (around opening edges)	–	–
	BU7	145.3	133.2	140 × 80	0.79	4D22	2D12	150	6	0.47	6D12 (*µ*_*av*_ = *µ*_*ah*_ = 2.4%) (around opening edges)	D10@ 35 mm (*ρ*_*va*_ = 5.6%)	–
	BU8	145.3	133.2	80 × 80	0.79	4D22	2D12	100	6	0.71	6D12 (*µ*_*av*_ = *µ*_*ah*_ = 2.4%) (around opening edges)	–	–

To ensure a shear failure mode, the lower reinforcement of all tested deep beams consists of double-layered four deformed bars with a diameter of 22 mm (*ρ*_*t*_ = 3.38% for group I and *ρ*_*t*_ = 5.43% for group II). The compression reinforcement of the first and second groups consists of two deformed bars with a diameter of 16 mm and 12 mm, respectively. The horizontal web bars were similar for all tested beams, which consisted of two deformed bars with a diameter of 10 mm on each beam side. Provided stirrups of the beams (vertical web reinforcement) of group I consist of plain bars of diameter 8 mm, while the provided stirrups of the beams of group II consist of plain bars of diameter 6 mm. The vertical web reinforcement ratios (*ρ*_v_%) were changed by varying the distance between the stirrups (*s*_*v*_) or by varying the diameter of the plain stirrup bars (*d*_*v*_), as shown in Table 1.

### Studied parameters

The experimental program involved the following examined parameters:

- Additional reinforcement techniques around openings: Three techniques were studied. Technique one: Additional vertical and horizontal bars around openings were used in BU3, BU6, and BU8 (with additional horizontal and vertical reinforcement percentages of *µ*_*av*_ = *µ*_*ah*_ = 2.7% for BU3, and *µ*_*av*_ = *µ*_*ah*_ = 2.4% for BU6 and BU8). Technique two: Additional stirrups and vertical and horizontal bars around openings were utilized in BU4 and BU7 (with additional stirrups percentages of *ρ*_*va*_ = 4.2% and 5.6%, respectively). Technique three: Additional cross bars around openings were used in BU5 (with additional horizontal bars percentages of *µ*_*ah*_ = 2.7% and additional side cross bars percentage of *µ*_*ax*_ = 1.8%), as shown in Figs. 2 and 3.
- Opening sizes: Beams BU2, BU3, BU4, and BU5 each had an opening of 150 mm x 150 mm (30% of the total beam height and 50% of shear span). Meanwhile, beams BU6 and BU7 had an opening of size 80 mm x 140 mm (20% of the total beam height and 51% of the shear span). BU8 had an opening measuring 80 mm x 80 mm (20% of the total beam height, 29% of the shear span). All the opening locations were in the shear span of the tested beam, and cut the line passing between the load and the support. The cross-section of the beams of group I was 100 mm x 500 mm, while that of group II was 80 mm x 400 mm, as shown in Fig. 1.
- Shear span to depth ratio (*a/d*): Group I tested with *a/d* ratio of 0.61, while Group II tested with *a/d* ratio of 0.79.

## UHPFRC mix

### Mix proportions

In the UHPFRC mix, CEM-I Portland cement grade 52.5 was utilized in accordance with European standards EN1971^48^, exhibiting a fineness of 345 m^2^/kg. The fine aggregate utilized in this investigation was natural siliceous sand with a particle size of 0/4 and a specific gravity of 2.65, in addition to quartz powder. Silica fume powder with a mean grain size of about 0.15 mm and a specific surface area of 19,000 m^2^/kg was incorporated into the mixture to fill the voids between cement and sand particles. The steel fibers utilized in this study were round-wavy fibers measuring 1 mm in diameter and 27.5 mm in length. The tensile strength of the fiber has been verified using a direct tension test, giving a yield strength of 1261 MPa and a tensile strength of 2441 MPa. The ratio of steel fibers utilized in all tested beams has been maintained at 1.5%. Superplasticizers (SP) derived from polycarboxylates, conforming to ASTM C494 type F^49^, were employed to maintain the concrete’s desired workability and ensure efficient consolidation. Table 2 shows the proportions for one cubic meter of UHPFRC mixture. UHPC specimens were fabricated similarly to those described in prior investigations^22,41^. The mixture components were integrated using a high-speed blender for ten minutes. Initially, sand and powders (cement, SF, and QP) were blended for two minutes at low speed; 50% of the water and SP were then incorporated, with low-speed mixing for an additional two minutes. Subsequently, the residual water and SP were incorporated and blended for four minutes. After that, steel fibers were included, and the blend persisted for another two minutes. UHPFRC samples were cured in water for the initial 24 h at room temperature (21 ± 2 °C). The beams were immersed in water for curing following demolding until the day of testing.

**Table 2:** Proportions of UHPFRC mixture (kg/m^3^).

Cement	Silica fume	Sand	Quartz powder	Water	Super plasticizer	Steel fibers
900	225	775	270	168	36	120

### Mechanical properties of UHPFRC mix

The mechanical properties of UHPFRC used in the deep beam tests were characterized through a comprehensive series of standardized laboratory tests^50–53^. All specimens were cast and tested on the same day as the beams were tested, following identical curing conditions to ensure consistent material behavior. The tests included cubes measuring 50 mm^50^, cylinders measuring 50 mm × 100 mm^51,52^, and beams measuring 50 mm × 50 mm × 200 mm^53^ for compressive, splitting tensile, and flexural strength evaluation, respectively. The average compressive strength obtained from the cube tests was *f*_*cu*_ = 145.30 MPa, while the average compressive strength obtained from the cylinder tests was *f*_*c*_^*’*^ = 133.20 MPa. The splitting tensile strength was recorded by compressing cylinders along their length as *f*_*sp*_ = 16.10 MPa, and the flexural strength from three-point bending for beams was *f*_*r*_ = 24.38 MPa. The mechanical characteristics of the UHPFRC mix are shown in Table 3.

**Table 3:** Mechanical properties of concrete mixture.

Mix ID	Cube compressive strength f_cu_ (MPa) ^50]^	Cylinder compressive strength f_c_^’^ (MPa) ^[51]^	Tensile strength f_sp_ (MPa) ^[52]^	Flexural strength f_*r*_ (MPa) ^[53]^
UHPFRC	145.30	133.20	16.10	24.38

### Reinforced steel bars

Reinforcement used in the tested deep beams was high-yield deformed steel for bars of 10 mm, 12 mm, 16 mm, and 22 mm in diameter, while mild steel was used for 6 mm and 8 mm bars in diameter. The mechanical properties of the reinforcement bars were determined through tensile testing of three samples for each bar size in accordance with ISO 15630-1:2010 standard^54^, to ensure conformity with design assumptions and experimental accuracy. The mean yield and ultimate strength of different bars are shown in Table 4.

**Table 4:** Mechanical properties of reinforcing steel.

D6		D8		D10		D12		D16		D22	
*f*_*y*_ (MPa)	*f*_*u*_ (MPa)	*f*_*y*_ (MPa)	*f*_*u*_ (MPa)	*f*_*y*_ (MPa)	*f*_*u*_ (MPa)	*f*_*y*_ (MPa)	*f*_*u*_ (MPa)	*f*_*y*_ (MPa)	*f*_*u*_ (MPa)	*f*_*y*_ (MPa)	*f*_*u*_ (MPa)
389.3	457.1	407.7	473.2	466.1	593.2	486.5	616.9	486.5	631.7	493.6	652.1

## Test set-up

**Fig. 4:**
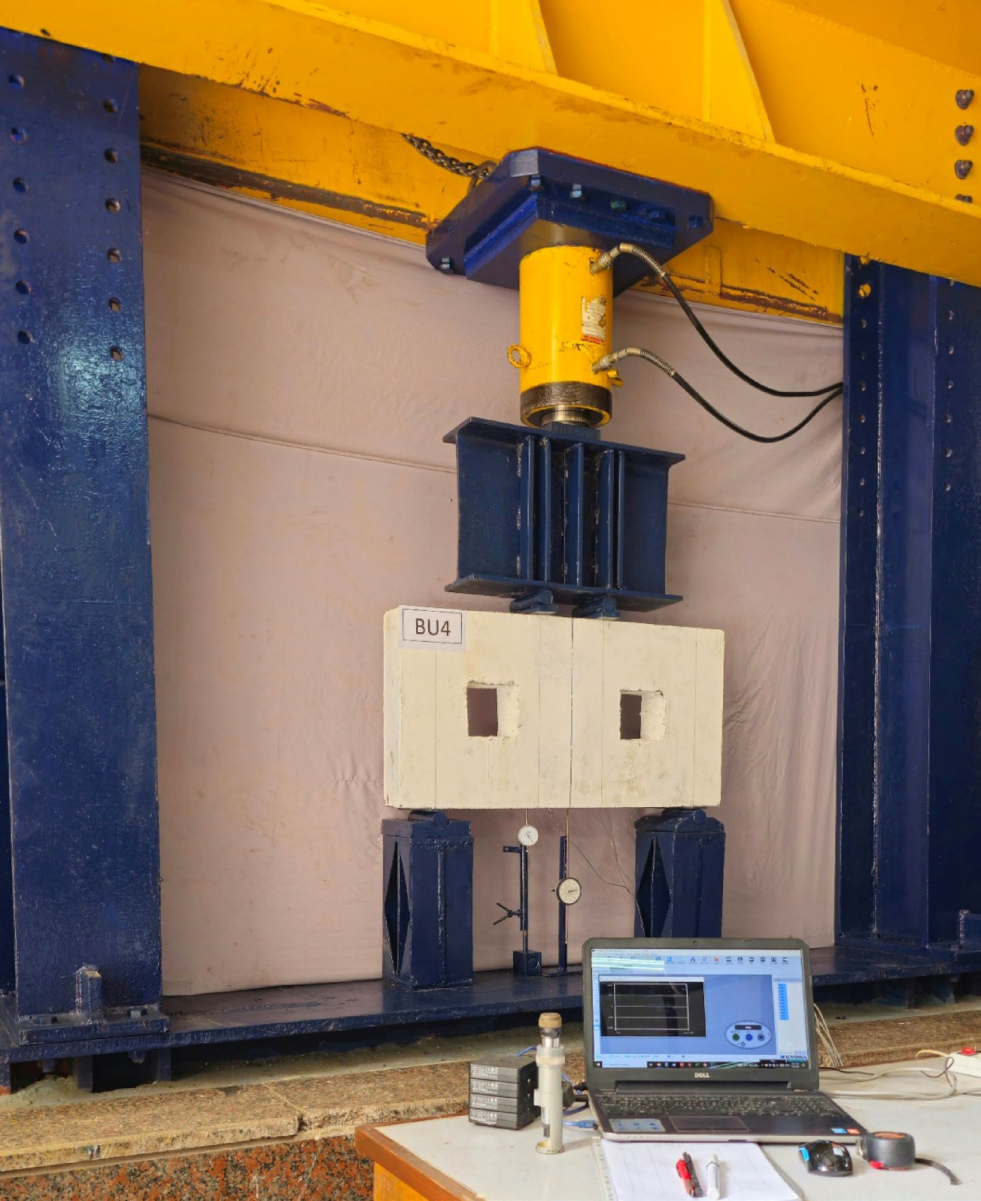
Photograph of the general view of the experimental setup.

As demonstrated in Fig. 4, the specimens were simply supported and tested under four-point loads using a 200-ton-capacity loading frame in the heavy structures laboratory of the faculty of engineering, Mansoura University. Similar conditions were maintained during casting and curing of each tested deep beam. To facilitate crack detection, the beams were painted white after being left to dry for two days before testing. To measure the beams’ deflection, two dial gauges with an accuracy of 0.01 mm and a capacity of 20.0 mm were placed at the midspan and beneath the loading point. A crack-detection microscope with an accuracy of 0.02 mm was used to measure the crack width. Electrical strain gauges, as seen in Fig. 4, were used to measure the strain of longitudinal bars, stirrups in the shear zone, horizontal web bars, and additional bars and stirrups around openings. Epoxy-based strain gauges measuring 10 mm in length, with an electrical resistance of 120 ohms, were employed. The load-controlled approach was implemented at a rate of 20 KN/min. After each load increment, all displacement and strain values were recorded. Crack progress was tracked and marked, and the breadth of cracks in the shear span region was documented.

## Experimental results

### Shear capacity and crack pattern

**Fig. 5:**
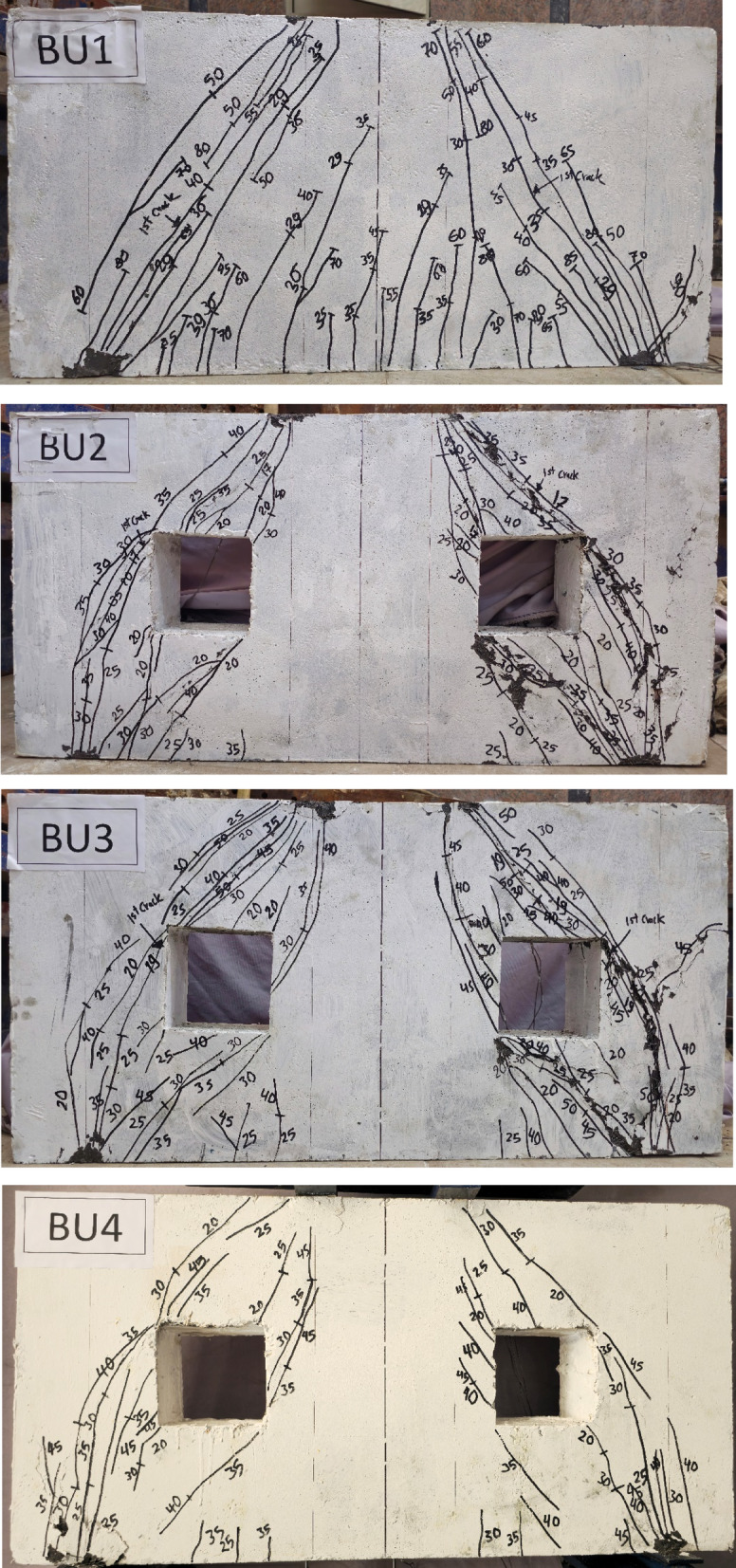
Photographs of failure modes of beams BU1, BU2, BU3, and BU4.

**Fig. 6:**
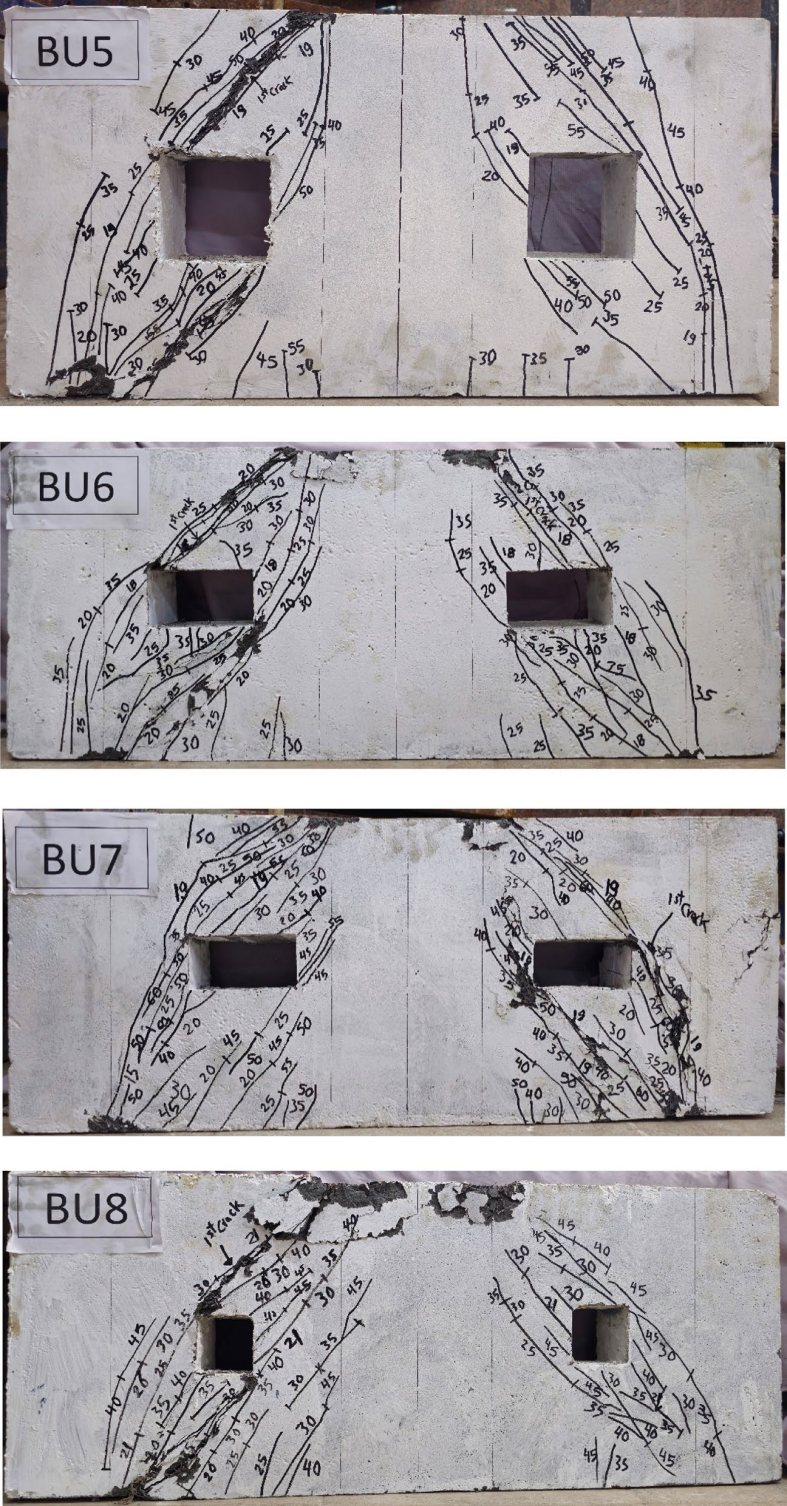
Photographs of failure modes of beams BU5, BU6, BU7, and BU8.

Table 5 presents the failure modes and the test results of the examined deep beams. Also, the photographs in Figs. 5 and 6 show the failure patterns for each specimen. Shear failure dominated across all the tested deep beams, with and without openings. For the control solid specimen BU1 of Group I, the first diagonal shear crack was observed at an approximate load of 100 kN (about 30% of the ultimate failure load). Steel fibers provide post-cracking tensile resistance via fiber bridging mechanisms, functioning as distributed micro-reinforcement. The delay in the first crack appearance is a key benefit of UHPFRC, as it extends the elastic behavior range and enhances serviceability performance under working loads. The principal diagonal shear crack continued to elongate and widen as the applied force increased, reaching a maximum load (2 V) of 970 kN, followed by new small diagonal cracks that appeared adjacent to the primary crack and aligned with the compression strut. The numerical markings on the concrete surface show the crack-propagation sequence and likely indicate different loading stages (2 V in tons) during testing. As loading reaches 70–80% of the ultimate capacity, the crack pattern becomes more intense with higher crack density and increasing crack widths, as shown by markings in Figs. 5 and 6. After all, shear failure happened due to concrete failure along the compression strut that lengthened between the loading point and supporting plate (in one of the two shear spans), as displayed in the photograph of specimen BU1 in Fig. 5.

**Table 5:** Test results.

Group	Beam	a/d ratio	2 V_crs_ kN	2 V_u, exp_ kN	V_crs_ kN	V_u, exp_ kN	$\frac{{V_{{crs}} }}{{V_{{u\,\,\exp }} }}$	$\frac{{V_{{u,\exp }} }}{{b.d\sqrt {f_{c}^{'} } }}$	Failure mode
I	BU1	0.61	290	970	145	485.0	0.30	0.93	Diagonal shear compression
	BU2	0.61	170	455	85	227.5	0.37	0.44	Shear failure at opening
	BU3	0.61	190	530	95	265.0	0.36	0.51	Shear failure at opening
	BU4	0.61	220	770	110	385.0	0.29	0.74	Shear failure at opening
	BU5	0.61	190	570	95	285.0	0.33	0.55	Shear failure at opening
II	BU6	0.79	180	410	90	205.0	0.44	0.63	Shear failure at opening
	BU7	0.79	190	665	95	332.5	0.29	1.03	Shear failure at opening
	BU8	0.79	210	475	105	237.5	0.44	0.73	Shear failure at opening

The specimen BU2 (with square openings of 150 mm x 150 mm and no additional opening reinforcement) cracked soon in shear at the corners of the openings aligned with the compression strut at an applied load of 170 kN (37.0% of the total ultimate load). As the applied force increased, the main shear cracks consistently extended from the corners of the opening to the support and from the upper corners to the loading plate, leading to total shear failure. The cracking load of BU2 decreased by 41.4% compared to that of the solid beam BU1. Additionally, the ultimate load of BU2 decreased by 40.4% compared to BU1, highlighting the significant impact of the existence of openings in the shear span. Figures 5 and 6, which show failure pattern photographs of the examined deep beams, demonstrate that one of the shear spans above and below the opening failed due to significant concrete fractures within the shear span.

For beams with provided additional reinforcement around openings in group I, the horizontal and vertical bars around openings (technique one) added in BU3 with percentages *µ*_*av*_ = *µ*_*ah*_ = 2.7%, postponed crack initiation by 11.8%, and increased ultimate shear capacity by 16.5%, compared to beam BU2 without additional reinforcement. Additional stirrups and vertical and horizontal bars (technique two) that were added in BU4 with percentages *µ*_*av*_ = *µ*_*ah*_ = 2.7% and *ρ*_*va*_ = 4.2% postponed crack initiation by 29.4%, and increased ultimate shear capacity by 69.2% compared to the beam BU2. Using additional cross bars around openings (technique three) in beam BU5, with percentages *µ*_*ax*_ = 1.8% and *µ*_*ah*_ = 2.7%, postponed crack initiation by 11.8% and increased ultimate shear capacity by 25.3% compared to the beam BU2. Although the percentage of additional side cross bars in BU5 (*µ*_*ax*_ = 1.8%) is lower than the percentage of additional vertical bars in BU3 (*µ*_*av*_ = 2.7%), the ultimate load of BU5 exceeds that of BU3 by nearly 8.8%. This counterintuitive result underscores the superior effectiveness of the side cross bars in arresting diagonal tension cracks. For the beams of group II, by comparing BU6 and BU7, the additional stirrups over and under the opening postponed crack initiation by 5.6%, and increased ultimate shear capacity by 62.3%. By comparing BU6 and BU8, which have the same additional reinforcement ratio (*µ*_*av*_ = *µ*_*ah*_ = 2.4%), the results showed that when the opening width decreased from 140 mm (51% of shear span) to 80 mm (29% of shear span), the first crack initiation was postponed by 16.7% and the ultimate shear capacity increased by 15.9%. Generally, the results of group II showed nearly the same trend as group I for the same technique in improving ultimate shear capacity, delaying crack formation, and reducing the main crack width under equivalent applied shear load, with a slight difference.

**Fig. 7:**
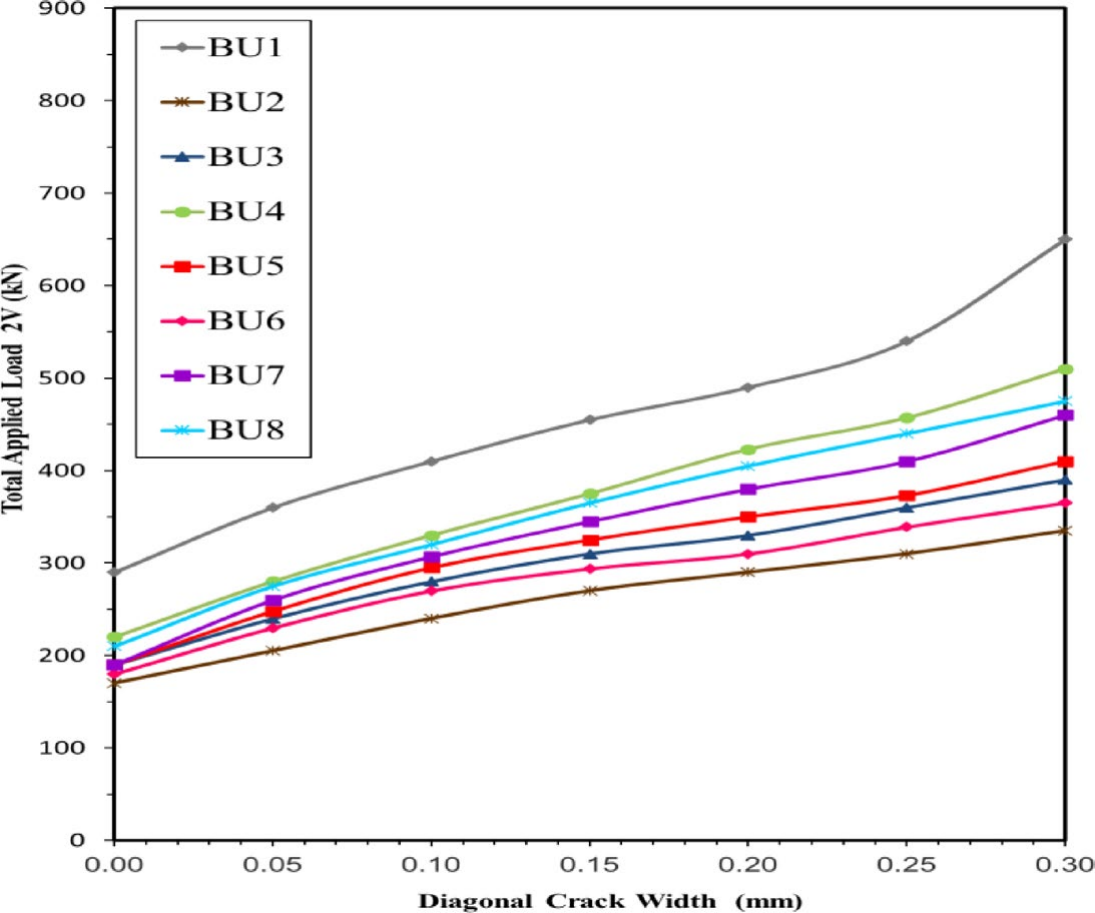
Applied load-diagonal crack width for tested specimens.

Figure 7 shows graphs of the total applied shear force (2 V) versus the width of the main shear crack for the two tested groups. It shows that the additional reinforcement in BU3, BU4, and BU5 reduces the main crack width at the same applied shear load. Additional stirrups and vertical and horizontal bars (technique two) in BU4 produce the least main crack width under equivalent applied shear load among the different tested techniques.

### Load-displacement response

**Fig. 8:**
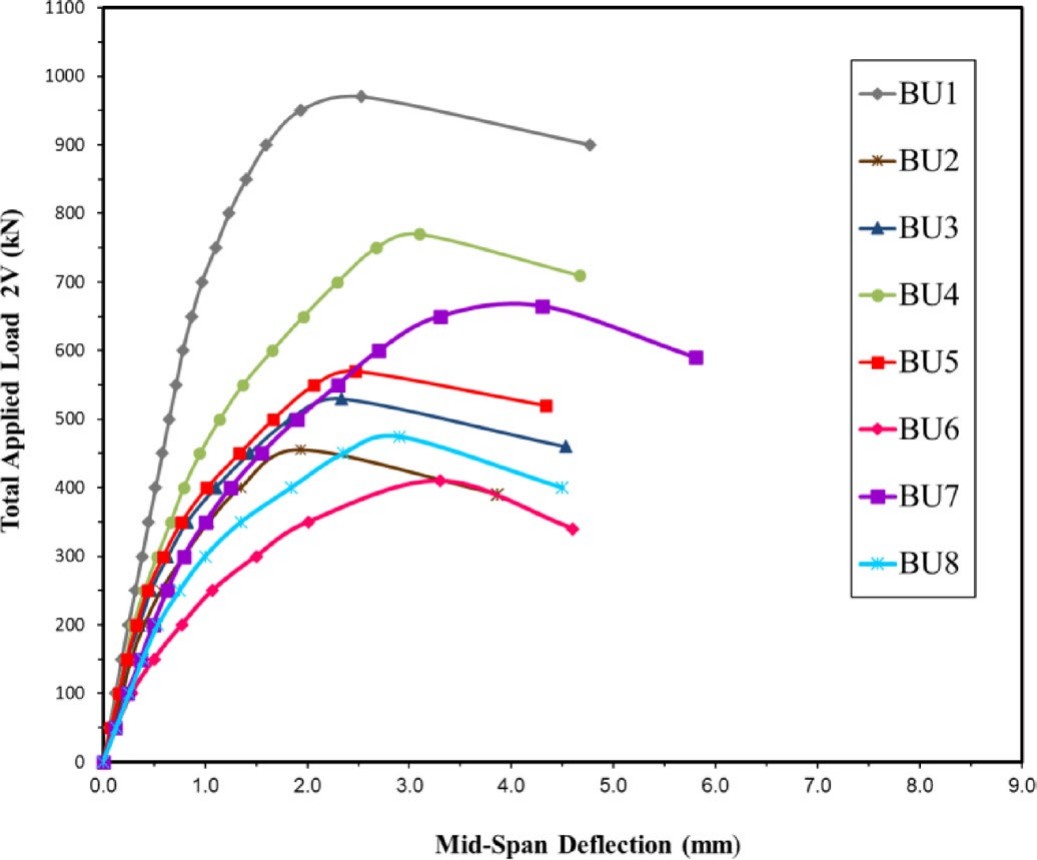
Applied load-midspan deflection for UHPFRC deep beams.

Figure 8 illustrates curves of the tested specimen’s measured total load (2 V) against mid-span deflection. The load-deflection response of the tested UHPFRC deep beams exhibited characteristic nonlinear behavior comprising three distinct phases: an initial, almost linear-elastic portion, indicating high initial stiffness. This elastic behaviour reflects the dense microstructure of UHPFRC. During this phase, the beams remain uncracked and exhibit minimal deflection relative to the applied load. After that, a transitional zone marked by progressive cracking and stiffness degradation, where deflection increases significantly once diagonal cracks develop. Finally, a post-peak softening phase happened, demonstrating the strain-hardening capacity of the fiber-reinforced matrix.

For beams of group I in Fig. 8, the beam BU1 exhibited the highest total ultimate load and highest stiffness, with a load capacity of 970.0 kN and ultimate mid-span deflection of 2.53 mm, highlighting the significant strength afforded by a solid web. Introducing openings without additional reinforcement, as in BU2, resulted in notable reductions in peak load and stiffness and an increase in deflection by nearly 190.0% compared to the solid beam at the same applied load. When straight bars were added around the opening edges in BU3, some improvement in stiffness was observed, and a decrease in deflection by 24.1% compared to BU2, but overall capacity remained slightly lower than that of the reference beam BU1. However, combining straight bars with vertical stirrups over or around the openings in BU4 led to a visible enhancement in post-cracking stiffness and higher load capacities, a decrease in deflection by 50.0% compared to BU2, confirming that stirrups significantly improve the effectiveness of the strut-and-tie mechanism and restrain crack growth. Cross bars, as employed in BU5, increased load capacity and reduced deflection by 27.6% compared to BU2, with an intermediate response curve compared to the reference beam. For beams with varying opening sizes in group II (Fig. 8), BU7, which incorporates the combined approach of straight bars with vertical stirrups around openings, exhibited the highest load capacity and reduced deflection at the same ultimate load. BU8, with smaller opening dimensions, showed reduced deflection and increased ultimate load compared to BU6, which has a larger opening size.

### Reinforcement strains

#### Strain of the vertical web reinforcement

**Fig. 9:**
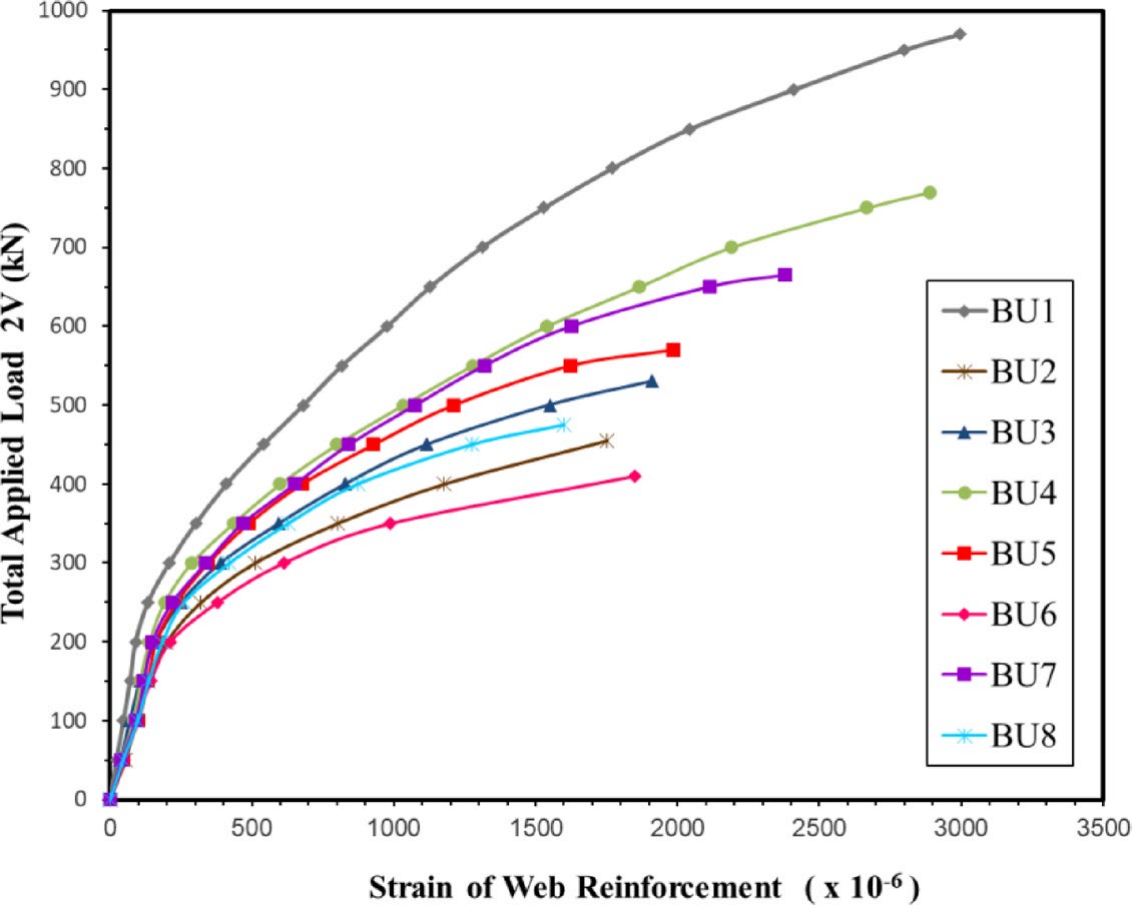
Measured strains in web reinforcement of UHPFRC deep beams.

Figure 9 shows the recorded strains of the vertical web reinforcement bar placed adjacent to the opening and intersecting the load path relative to the total applied load (average of G3 and G6). The strain is recorded at the center of the vertical leg. Generally, adding different strengthening techniques around openings increases the recorded strain at the same applied load. The strain in the vertical web bars was minimal before the formation of the main diagonal crack. After cracks appear, the strain increases noticeably as the diagonal shear crack widens until the concrete fails. Only the vertical web bars in the reference beam BU1 and beams with additional stirrups, such as BU4 and BU7, reach the yield stress just before failure, while the main stirrups in other specimens did not reach the yield point at failure, as shown in Fig. 9. This is due to the high ultimate load recovery provided by the additional stirrups. The introduction of web openings (BU2) reduced strains, reflecting the disruption of the direct compression strut and necessitating alternative load paths around the opening perimeter.

#### Strain of the vertical web reinforcement

The tensile strain in the middle of the bottom longitudinal steel bars steadily increases with each load increment. Upon initiation of diagonal shear cracks, the strain rate in main bars increased as these bars became integral components of the internal truss system, carrying horizontal tension forces to equilibrate inclined compression struts. All beams of Group I reach the ultimate load without yielding the main bottom bars due to shear failure in the compression strut and the high yielding stress of the steel bars. The additional bars in strengthened beams have a minimal impact on the recorded strain of the bottom longitudinal reinforcement compared to those without additional bars. Group II beams exhibited significantly lower reinforcement strains due to the higher *a/d* ratio (0.79) and smaller depth, which fundamentally changed stress flow patterns and reduced the contribution of horizontal web reinforcement to overall shear resistance, with load transfer increasingly dominated by direct compression strut action.

### Ductility

**Fig. 10:**
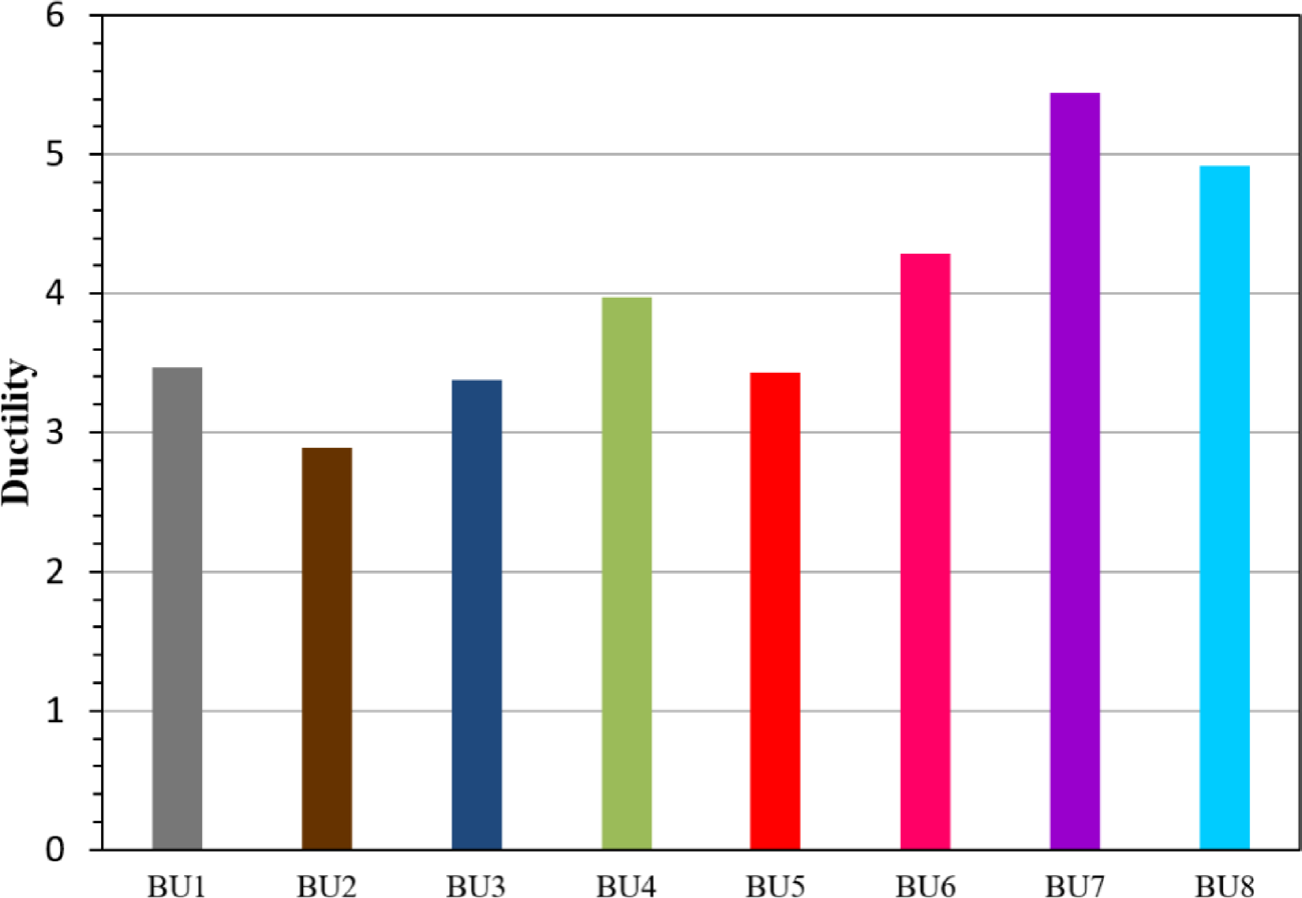
Ductility index for the tested specimens.

The ductility index, defined as the ratio of ultimate to yield deflection, was used to quantify the deformation capacity of the tested beams. Compared with the solid reference beam BU1, introducing an unreinforced 150 × 150 mm opening in BU2 reduced ductility by 16.5%, as shown in Fig. 10. The straight bars positioned around the opening edges (with percentages *µ*_*av*_ = *µ*_*ah*_ = 2.7%) added in BU3 increased the Ductility by 16.8% compared to the unreinforced opening beam. The combination of straight bars around edges (with percentages *µ*_*av*_ = *µ*_*ah*_ = 2.7%) with closely spaced stirrups positioned over and under the opening (with percentage *ρ*_*va*_ = 4.2%) in BU4 enhanced ductility by 37.4% compared to BU2. This ensures the importance of stirrups in improving ductility. Using additional cross bars around openings in beam BU5 (with percentages *µ*_*ax*_ = 1.8% and *µ*_*ah*_ = 2.7%) increased ductility by 18.6% compared to beam BU2. For group II beams, comparing BU6 and BU7, the additional stirrups over and under the opening improved ductility by 27.0%. By comparing BU6 and BU7, which have the same ratio and configuration of additional reinforcement, changing the opening width from 140 mm to 80 mm increased ductility by 14.7%. This showed that reducing the opening size improved ductility response.

### Effect of different additional reinforcement techniques around openings

**Fig. 11:**
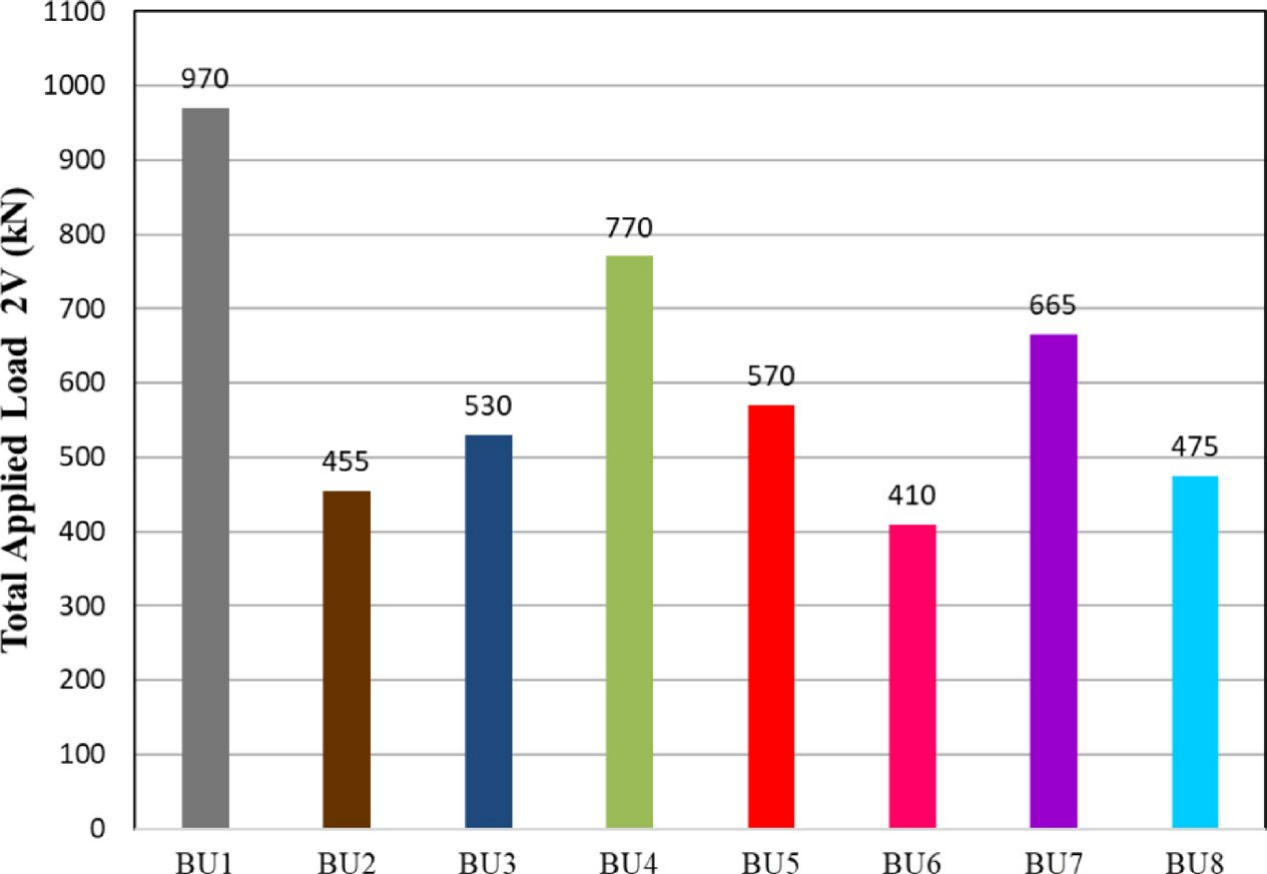
Specimens’ total ultimate loads.

The previous results of this experimental investigation thoroughly demonstrated that additional reinforcement around web openings in UHPFRC deep beams significantly and multifacetedly influences shear capacity, deformation characteristics, strain distribution, and crack pattern. Figure 11 shows the total ultimate loads for the tested specimens. The beams of group I feature a 150 × 150 mm opening (30.0% of the total height and shear span), which is relatively large and significantly disrupts the strut-and-tie mechanism. The beam BU2, without additional reinforcement around openings, exhibited substantial degradation in ultimate shear capacity with a reduction of 53.1% relative to the solid reference beam BU1, reflecting severe disruption of load transfer mechanisms and stress concentration at opening corners. Opening existence also accelerates crack formation by 41.4% compared to the solid beam. This degradation in ultimate shear capacity demonstrates that UHPFRC deep beams alone, without strategic additional reinforcement, are insufficient to compensate for large web openings in shear-critical regions.

- Technique one: The straight bars positioned around the opening edges (with percentages *µ*_*av*_ = *µ*_*ah*_ = 2.7%) added in BU3 increased the ultimate load capacity by 16.5% compared to the unreinforced opening beam, demonstrating 54.6% recovery of the solid beam BU1 capacity. Additional straight bars positioned around the opening edges also delayed crack formation by 11.8% in BU3 relative to the unreinforced opening, indicating that straight bars alone provided a direct tension path that bypassed the opening perimeter but failed to restore the complete truss mechanism or establish load-path redundancy.
- Technique two: The combination of straight bars around edges (with percentages *µ*_*av*_ = *µ*_*ah*_ = 2.7%) with closely spaced stirrups positioned over and under the opening (with percentage *ρ*_*va*_ = 4.2%) in BU4 demonstrated exceptional effectiveness. This technique enhanced the ultimate load capacity by 69.2% compared to BU2 (beam without additional reinforcement) and recovered 79.4% of the solid beam BU1 capacity. Closely spaced stirrups applied in BU4 delayed the crack formation by 29.4% compared to the unreinforced opening. This remarkable increase in shear capacity indicates that the enhanced vertical confinement provided by closely spaced stirrups effectively delayed compression strut deterioration, controlled diagonal crack propagation from opening corners, and facilitated progressive load redistribution, transforming the failure mode from brittle strut crushing to a more ductile response approaching stirrup-yielding behavior. Additional vertical stirrups make the beam approach the reference beam’s structural behavior despite the substantial presence of a 150 × 150 mm opening, demonstrating that combined reinforcement strategies can nearly restore the load transfer mechanisms of solid beams.
- Technique three: The combined configuration, incorporating upper and lower straight bars with diagonal crossbars around the opening vertical sides (with percentages *µ*_*ax*_ = 1.8% and *µ*_*ah*_ = 2.7%) in beam BU5, represented an alternative approach. It achieved a 25.3% increase in shear capacity over unreinforced openings, demonstrating 58.8% of the respective solid beam capacity (BU1). It also delayed the crack formation by 11.8% compared to unreinforced opening. Diagonal crossbars provide alternative inclined tension paths, enabling a distributed fine-crack pattern characteristic of UHPFRC strain-hardening behavior rather than localized major cracks.

For beams of group II, by comparing BU6 and BU7, the additional stirrups over and under the opening postponed crack initiation by 5.6%, and increased ultimate shear capacity by 62.3%. Although the percentage of the straight bars positioned around the opening edges added in BU6 and BU8 was the same, the ultimate load of BU8 exceeded that of BU6 by 15.9%. This increase in the percentage of ultimate load among these beams arises from the opening dimensions, since BU6 features an 80 × 140 mm opening (20.0% of total height and 50.9% of shear span), while BU8 has an 80 × 80 mm opening (20.0% of total height and 29.1% of shear span). This demonstrates that the opening dimension is the primary factor influencing the ultimate shear capacity, with larger openings creating more severe disruptions to the natural load path.

Across both groups, the combined reinforcement approach (straight bars with stirrups) consistently demonstrated superior performance, effectively restoring load-transfer mechanisms that approach reference-beam behavior. The stirrup-enhanced configurations in BU4 and BU7 exhibited comparable structural responses despite belonging to different groups, indicating that this reinforcement technique provides reliable strengthening regardless of specific opening geometries, though the absolute magnitude of improvement remains contingent on opening size and position.

The results of this study are mainly specific to UHPFRC deep beams, as their enhanced tensile and post-cracking properties significantly influence shear transfer and crack control. Although previous studies that applied similar reinforcement configurations around openings in normal-strength concrete deep beams showed a similar trend, with differences in the percentage of improvement, direct quantitative extrapolation to normal-strength concrete deep beams should be approached with caution.

## Numerical modeling of UHPFRC deep beams

### Numerical model

**Fig. 12:**
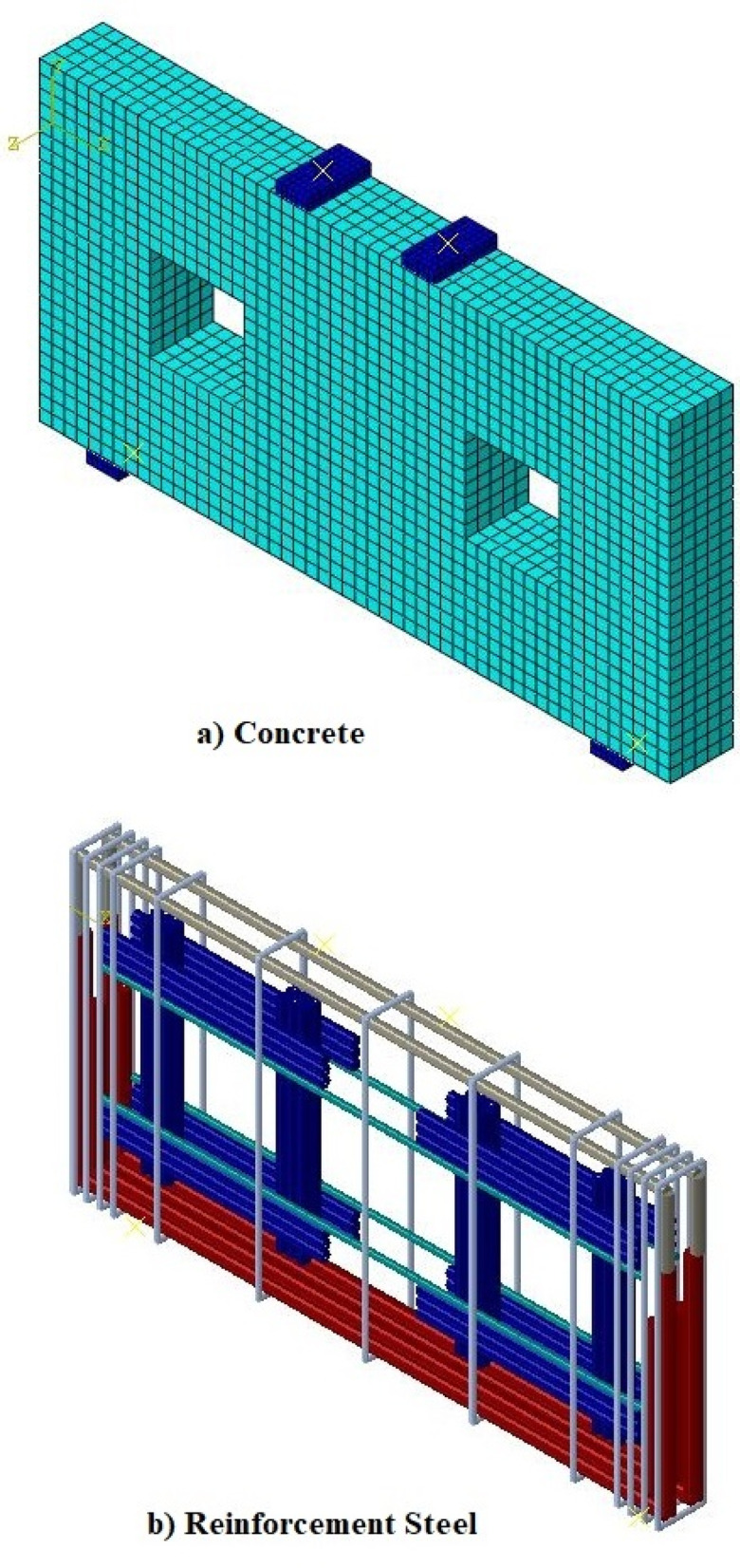
Meshing of UHPFRC deep beams.

**Fig. 13:**
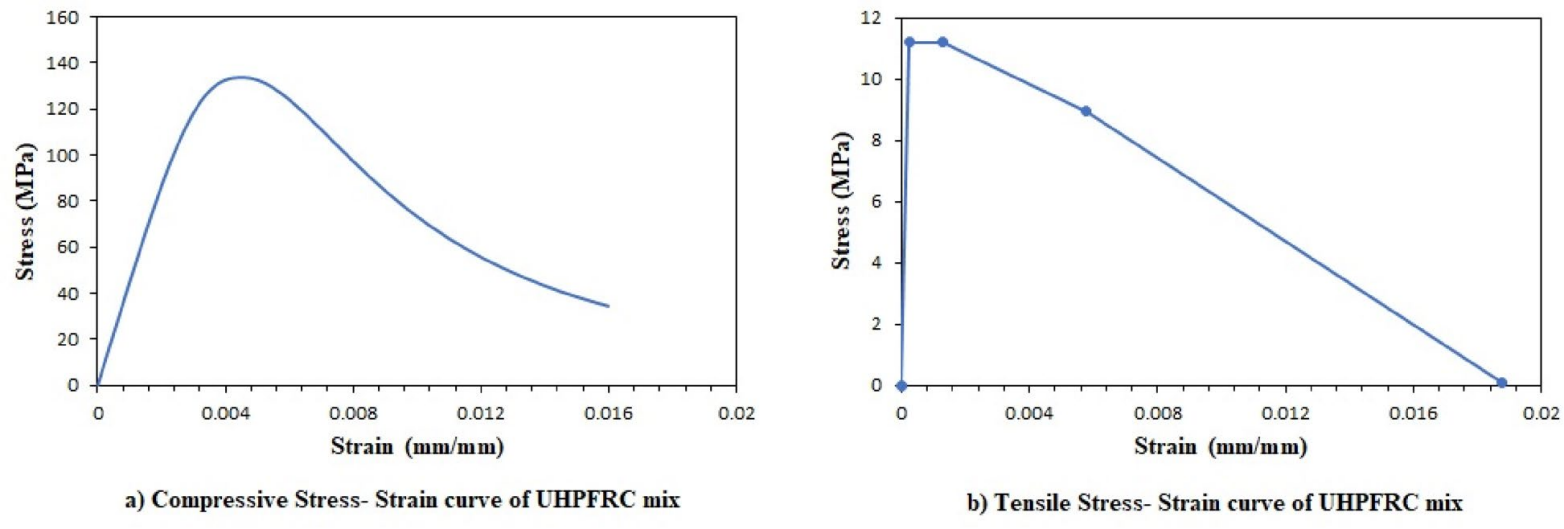
Compressive and tensile stress-strain curves of UHPFRC mix derived from AFGC.

A three-dimensional numerical model of UHPFRC deep beams was developed using the ABAQUS software^55^, incorporating the Concrete Damage Plasticity (CDP) model to characterize the material’s complex stress-strain response. The constitutive behavior of UHPFRC was characterized using the Drucker-Prager plastic flow function, with the following material parameters. The eccentricity (*ε*) parameter, which defines the curvature of the plastic potential surface in the meridional plane, was set to *ε* = 0.1. The shape factor (*K*_*c*_), which controls the three-dimensional asymmetry of the yield surface in deviatoric stress space, was set to 0.667. The dilation angle (*ψ*), governing the magnitude of plastic volumetric strain relative to plastic shear strain, was established at *ψ* = 30°. The stress ratio (*f*_*b0*_*/f*_*c0*_), representing the ratio of equibiaxial to uniaxial compressive yield stress, was specified as *f*_*b0*_*/f*_*c0*_ = 1.05. The viscosity parameter, a numerical regularization that introduces viscoplastic behavior to smooth the material’s softening response and aid convergence, was set to 0.0005. The concrete volume of the deep beam specimens was discretized using eight-node solid elements (C3D8R). The steel bars were explicitly represented as three-dimensional truss elements (T3D2). A mesh sensitivity analysis was performed to examine the influence of element size on the numerical response and to ensure that the selected discretization provided mesh-independent results. Four mesh sizes (10 mm, 20 mm, 30 mm, 50 mm) were examined on three of the tested beams (BU1, BU2, BU3). The comparison showed only minor differences in the predicted ultimate load and load-deflection response, as shown in Table 6, whereas the coarse mesh produced a slightly less accurate representation of the localized damage zone. Accordingly, a 20 mm mesh size was adopted in the present study because it offered a satisfactory compromise between agreement with experimental results and analysis time. The mesh utilized in the suggested numerical model is shown in Fig. 12. The compressive and tensile stress-strain curves of UHPFRC in the plastic range are shown in Fig. 13. These curves were derived from AFGC-2013^56^. The elastic modulus of UHPFRC was calculated using the AFGC-2013, implementing the equation [*E*_*c*_=9100×(*f*_*c*_*’*)^1/3^].

**Table 6:** Mesh sensitivity analysis results for BU2 Specimen.

Mesh size (mm)	Ultimate shear load		Ultimate displacement	
	V_u, Num_ (kN)	$\frac{{V_{{u,Exp}} }}{{V_{{u,Num}} }}$	∆_u, Num_ (mm)	$\frac{{\Delta _{{u,Exp}} }}{{\Delta _{{u,Num}} }}$
10	209.0	1.09	1.51	1.28
20	208.8	1.09	1.47	1.31
30	201.7	1.13	1.48	1.30
50	202.8	1.12	1.41	1.37

The steel bars were modeled as an elastic-perfectly plastic material using the isotropic hardening rule available in the ABAQUS material library. The elastic behavior of the steel was defined by assigning a modulus of elasticity (*E*_*s*_=2 × 10^5^ MPa) and a Poisson’s ratio of 0.30, which are characteristic values for steel bars. To establish a fully bonded interface between the steel reinforcement and the surrounding concrete matrix, the embedded element method (EEM) was implemented, in which truss elements are constrained to move in tandem with the host concrete elements. Boundary conditions were defined to simulate a typical simply-supported deep beam configuration, with one support designated as a hinged boundary condition (constraining all translational degrees of freedom: *u₁* = *u₂* = *u₃* = 0.0) and the opposite support configured as a roller boundary condition (permitting longitudinal and lateral translations while restricting vertical displacement: *u₂* = 0.0, with *u₁* ≠ 0.0 and *u₃* ≠ 0.0), thereby allowing for realistic load transfer mechanisms and rotational compatibility at the supports. To prevent penetration between the bearing plates and the deep beam surface, a general contact algorithm was implemented to automatically detect and manage surface-to-surface contact interactions throughout the analysis. The damage parameters for compression (*d*_*c*_) and tension (*d*_*t*_) were established to measure the decrease in stiffness noted in the descending segment of the stress–strain curve. The parameters were computed using the formula d = 1 - (actual strength / ultimate strength) for both *d*_*c*_ and *d*_*t*_. The values of dc and dt range from 0 to 1, with 0 indicating an undamaged condition and 1 signifying total failure, while intermediate values reflect varying degrees of partial damage.

### Comparison of experimental results with the numerical model

The developed nonlinear numerical model was validated by comparing its predictions with experimental results for UHPFRC deep beams in this study. The results show close agreement in both ultimate load and mid-span deflection. The validation mainly focused on the predicted ultimate load capacities, failure patterns, and the total load–midspan deflection response. Table 7 compares the experimentally reported ultimate shear load *V*_*u, EXP*_, and the numerically predicted ultimate shear load *V*_*u, NUM*_, as well as the corresponding experimental mid-span deflection *∆*_*u, EXP*_, and the numerical mid-span deflection *∆*_*u, NUM*_. The average ratios for group I and group II of *V*_*u, EXP*_ / *V*_*u, NUM*_ computed from Table 7 are equal to 1.08 and 1.09, respectively with total average ratio of 1.08, and the average ratios of *∆*_*u, EXP*_ / *∆*_*u, NUM*_ are equal to 1.27 and 1.36 respectively with total average ratio of 1.30, indicating that the proposed model predicts the shear behavior with good accuracy despite changes in the specimen section, opening size, and additional reinforcement techniques.

**Table 7:** Comparison between experimental and numerical results for the tested beams.

Beam	a/d	Ultimate shear load			Ultimate displacement			Failure mode	
		V_u, Exp_ (kN)	V_u, Num_ (kN)	$\frac{{V_{{u,Exp}} }}{{V_{{u,Num}} }}$	∆_u, Exp_ (mm)	∆_u, Num_ (mm)	$\frac{{\Delta _{{u,Exp}} }}{{\Delta _{{u,Num}} }}$	Experimental	Numerical
BU1	0.61	485.0	430.1	1.13	2.53	2.17	1.17	Shear comp.	Shear comp.
BU2	0.61	227.5	208.8	1.09	1.93	1.47	1.31	Shear at opening	Shear at opening
BU3	0.61	265.0	247.6	1.07	2.33	1.69	1.38	Shear at opening	Shear at opening
BU4	0.61	385.0	376.0	1.02	3.10	2.85	1.09	Shear at opening	Shear at opening
BU5	0.61	285.0	262.9	1.08	2.47	1.78	1.39	Shear at opening	Shear at opening
BU6	0.79	205.0	196.5	1.04	3.30	2.37	1.39	Shear at opening	Shear at opening
BU7	0.79	332.5	277.0	1.20	4.30	3.23	1.33	Shear at opening	Shear at opening
BU8	0.79	237.5	229.4	1.04	2.90	2.12	1.37	Shear at opening	Shear at opening

**Fig. 14:**
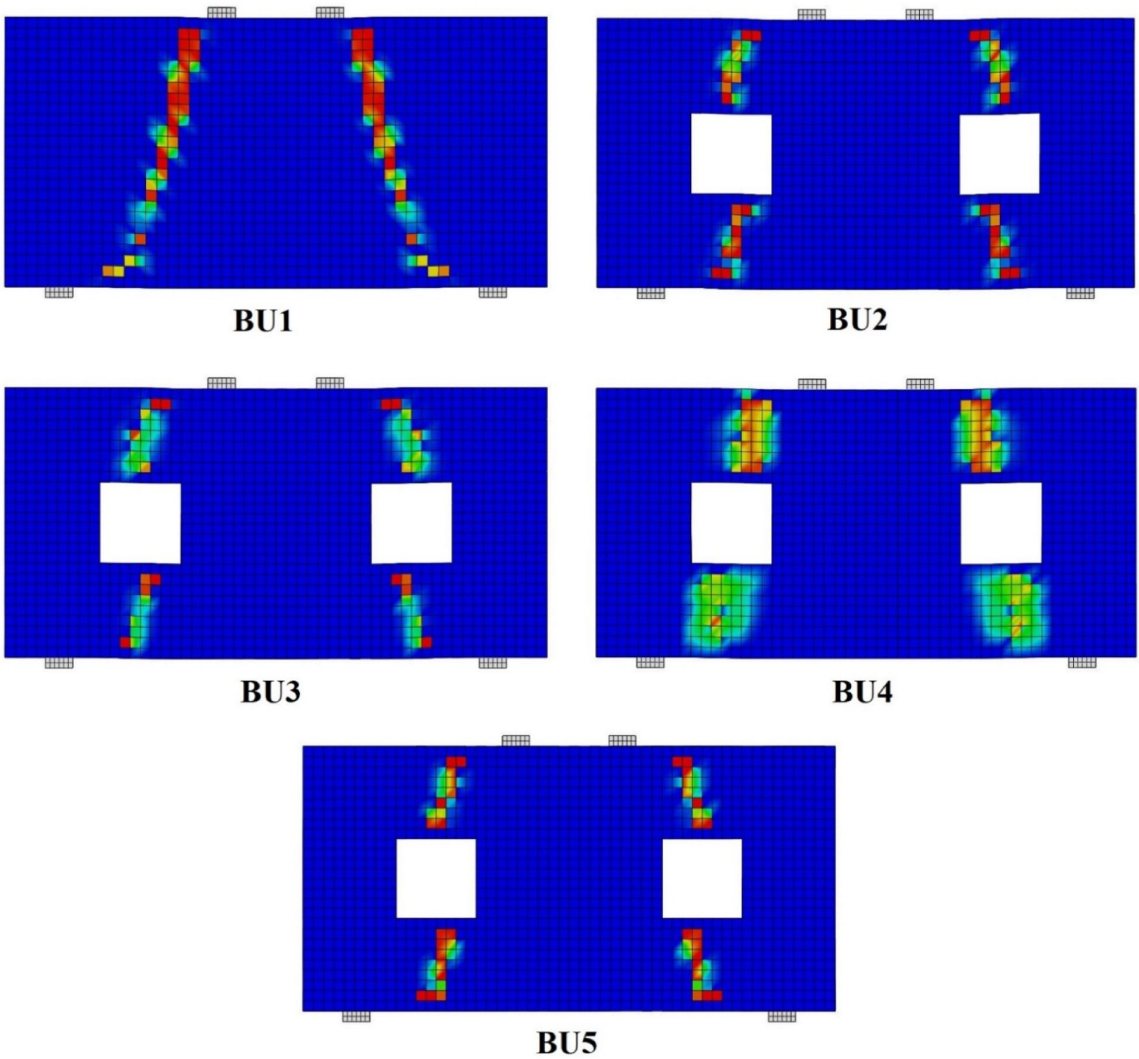
Numerical failure modes for beams of Group I.

**Fig. 15:**
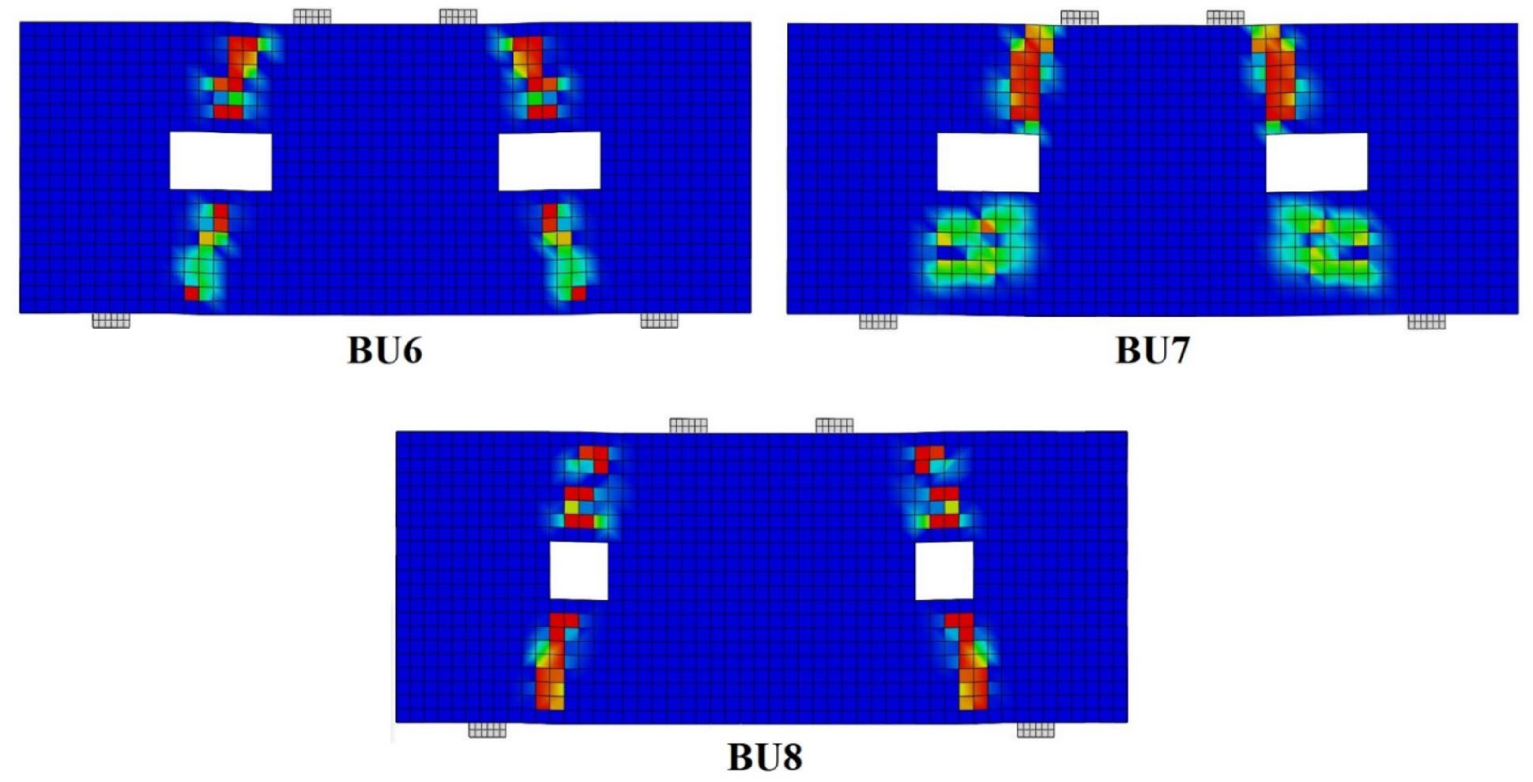
Numerical failure modes for beams of Group II.

**Fig. 16:**
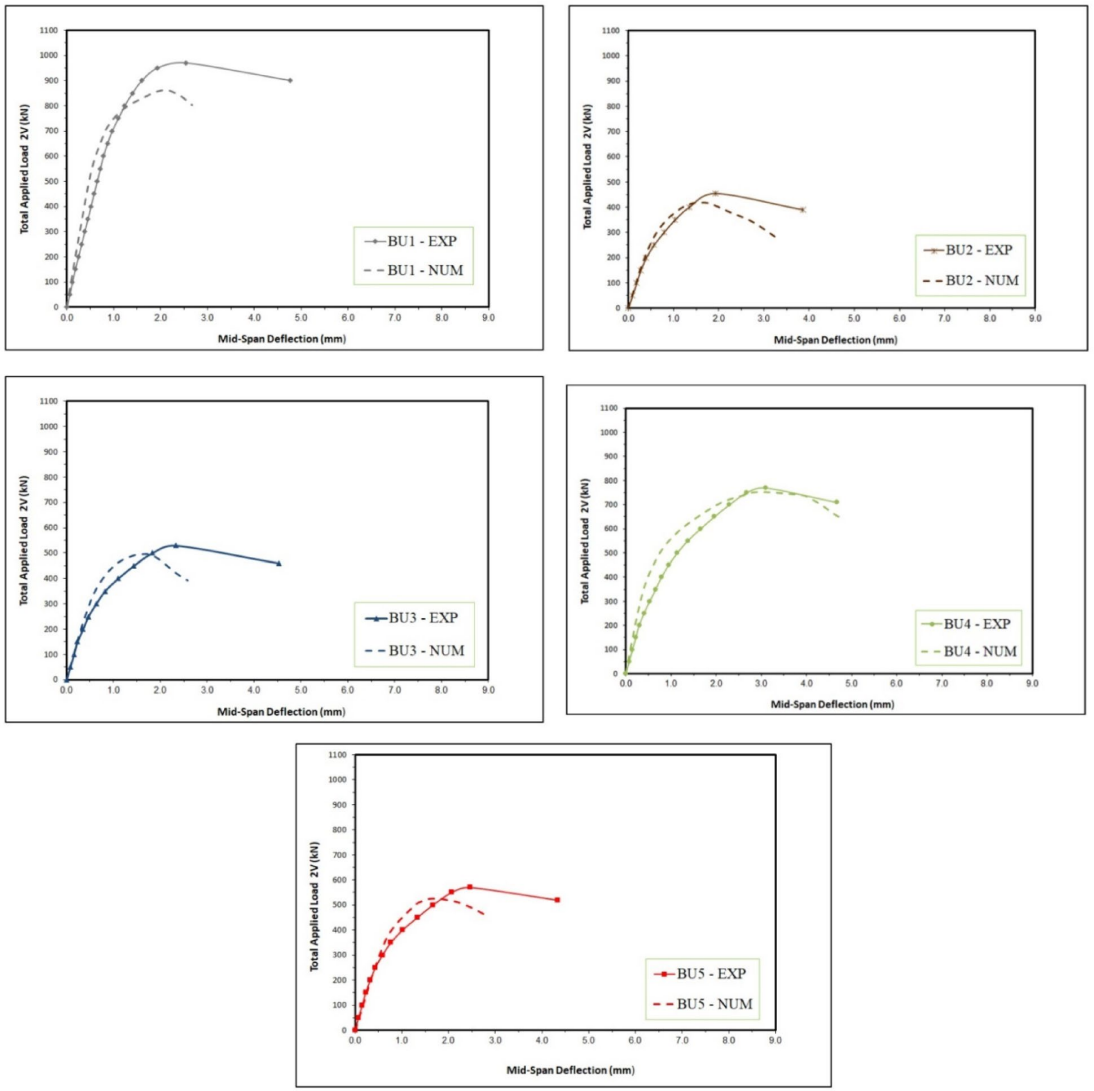
Experimental and numerical load-displacement curves for beams of Group I.

**Fig. 17:**
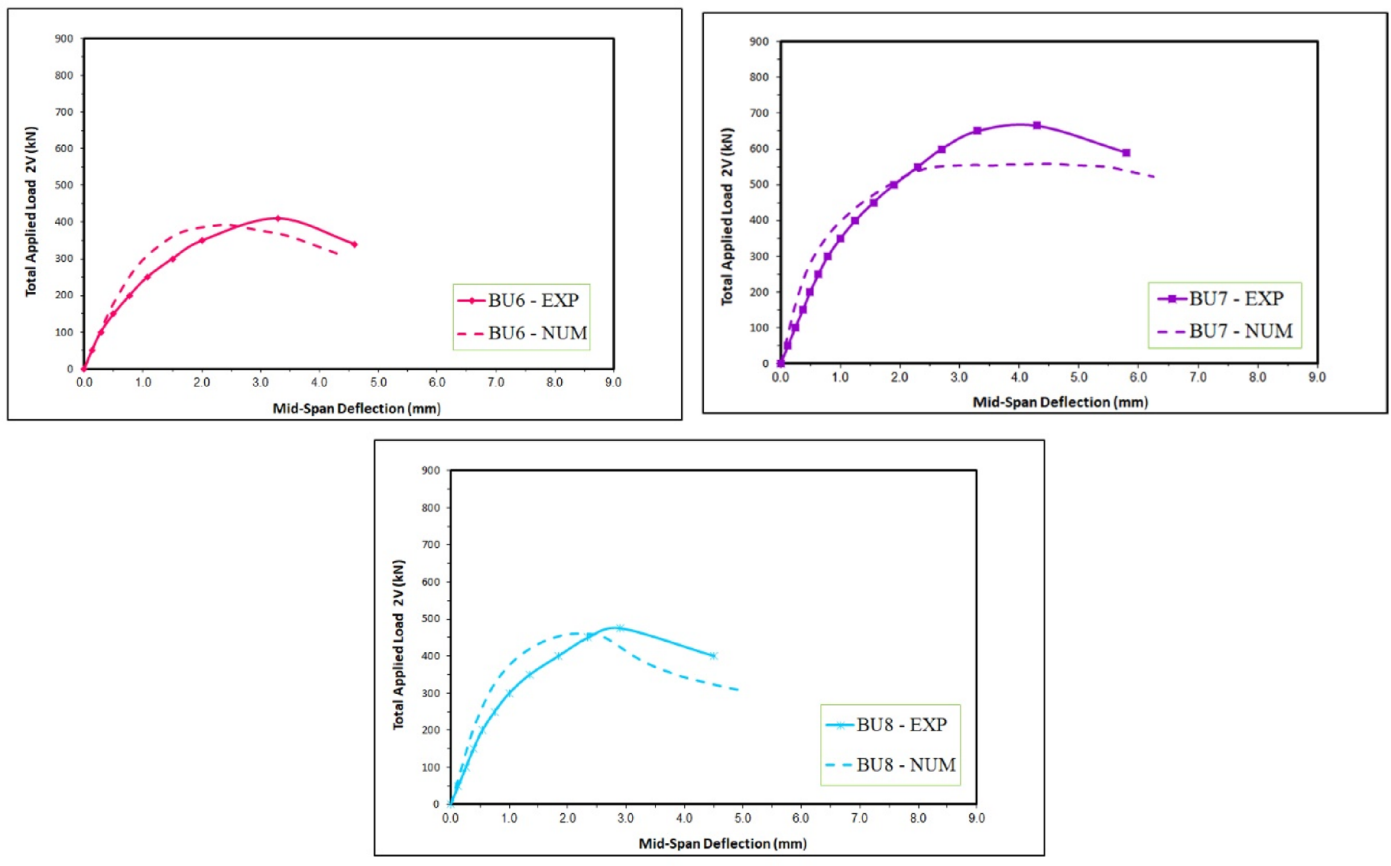
Experimental and numerical load-displacement curves for beams of Group II.

The predicted failure patterns for the tested beams in groups I and II are presented in Figs. 14 and 15, using a damage parameter to highlight the failed concrete regions. These numerical failure modes closely agree with the experimentally observed crack localization and dominant diagonal shear damage zones, when compared to Figs. 5 and 6 and to the failure modes reported in Table 5. Furthermore, the experimental and numerical ultimate load–mid-span curves for beams in Groups I and II are compared in Figs. 16 and 17, where the overall agreement is adequate, indicating that the proposed numerical model captures the global stiffness evolution and the peak/softening trends with satisfactory consistency. Overall, the strong agreement between numerical and experimental responses demonstrates that the proposed numerical model can reliably simulate the structural behavior of UHPFRC deep beams with different opening reinforcement ratios and shapes, opening sizes, and *a/d* ratios.

## Summary and conclusions

This study experimentally and numerically examined the shear response of simply supported UHPFRC deep beams with internal opening reinforcement, with emphasis on the effectiveness of internal additional reinforcement detailing along the opening perimeter. The investigated parameters were the additional reinforcement technique, opening size (30% or 20% of the total beam height), and the shear span-to-depth ratio *a/d* ( a ratio of 0.61 and 0.79) The tested internal opening techniques were straight bars around the opening (*µ*_*av*_ = *µ*_*ah*_ = 2.7% or 2.4%), straight bars (*µ*_*av*_ = *µ*_*ah*_ = 2.7% or 2.4%) combined with additional stirrups (*ρ*_*va*_ = 4.2% or 5.6%), and diagonal cross-bars (*µ*_*ax*_ = 1.8% and *µ*_*ah*_ = 2.7%). The beams were tested under four-point loading to promote shear behavior, and responses were assessed in terms of cracking load, ultimate shear capacity, load–deflection behavior, crack development, and reinforcement strain trends. This investigation permits the following conclusions:

1. Introducing an unreinforced opening markedly reduced shear capacity: the cracking load decreased by 41.4%, and the ultimate load decreased by about 40.4% relative to the corresponding solid beam.
2. Beams strengthened with straight vertical and horizontal bars around the opening (*µ*_*av*_ = *µ*_*ah*_ = 2.7%) exhibited improved performance. This reinforcement arrangement delayed cracking by 11.8% and increased the ultimate shear capacity by 16.5% compared with beams with unreinforced openings.
3. Beams reinforced with diagonal cross-bars around the opening (*µ*_*ax*_ = 1.8% and *µ*_*ah*_ = 2.7%) also showed enhanced behavior. This configuration delayed cracking by 11.8% and increased the ultimate shear capacity by 25.3% relative to the unreinforced opening beams.
4. Among the studied reinforcement techniques, the combined use of straight bars around the opening (*µ*_*av*_ = *µ*_*ah*_ = 2.7%) and additional stirrups above and below the opening (*ρ*_*va*_ = 4.2%) was the most efficient in delaying crack initiation and increasing the ultimate shear capacity by 29.4% and 69.2%, respectively, relative to the beam with unreinforced opening, indicating that confinement and load-path continuity are both critical for deep beams with openings.
5. The comparative recovery ratios relative to the solid beam confirm that perimeter bars alone are insufficient to fully compensate for the loss in shear capacity caused by the opening, whereas the addition of stirrups substantially improves structural integrity and increases the safety margin.
6. The proposed numerical model reliably simulates the shear behavior of UHPFRC deep beams with different opening reinforcement ratios and shapes, opening sizes, and *a/d* ratios.

The present investigation is constrained by the relatively limited number of specimens, the restricted range of opening geometries, *a/d* ratios, and the lack of environmental durability evaluation. Therefore, future studies should extend the current findings by examining additional parameters, particularly the long-term durability and structural performance of UHPFRC deep beams.

### Design recommendation

For UHPFRC deep beams with openings located in the shear span, the use of combined reinforcement detailing, particularly straight bars with closely spaced stirrups around the opening, is recommended to reduce capacity loss and improve load transfer efficiency.

### Recommendation for future research

- Future work should explore strut-and-tie modeling for UHPFRC deep beams with openings along the strut line, thereby providing simplified design guidelines that complement the experimental and numerical data presented herein.
- Future work should examine wider ranges of a/d ratio, opening size, and opening location to better understand their effects on load transfer and shear resistance.

## Data Availability

Data will be made available on request.

## Abbreviations

UHPFRC: Ultra-high-performance fiber-reinforced concrete
RC: Reinforced concrete
STM: Strut-and-tie method
RPC: Reactive powder concrete
CFRP: Carbon fiber-reinforced polymer
FRP: Fiber-reinforced polymer
NSM: Near-surface mounted
ECC: Engineered cementitious composites
TRC: Textile-reinforced concrete
WWM: Welded wire mesh
ACI: American concrete institute
EC: Eurocode
ECP: Egyptian code of practice
SP: Superplasticizers
CDP: Concrete damage plasticity
EEM: Embedded element method

## Notations

*a/d*: Shear span-to-depth ratio
2*V*: Total applied shear force
*ρ*_*t*_: Tension (bottom) longitudinal reinforcement ratio = *A*_*sl*_ / *b×d*
*ρ*_v_: Vertical web reinforcement ratio (stirrups) = *A*_*v*_ / *b*×*s*_*v*_
*s*_*v*_: Spacing between vertical stirrups
*d*_*v*_: Diameter of vertical stirrups
*μ*_*av*_: Ratio of additional vertical reinforcement around openings = *A*_*sva*_ / *b×d*
*μ*_*ah*_: Ratio of additional horizontal reinforcement around openings = *A*_*sha*_ / *b×d*
*μ*_*ax*_: Ratio of additional cross reinforcement around openings = *A*_*sxa*_ / *b×d*
*ρ*_*va*_: Ratio of additional stirrups over and under the opening = *A*_*va*_ / *b×s*_*va*_
*f*_*cu*_: Cube compressive strength of concrete
*f*_*c*_^*'*^: Cylinder compressive strength of concrete
*f*_*sp*_: Splitting tensile strength of concrete
*f*_*r*_: Flexural strength of concrete
*E*_*c*_: Modulus of elasticity of UHPFRC
*ε*: Eccentricity parameter in CDP
*K*_*c*_: Shape factor in CDP
*ψ*: Dilation angle in CDP
*f*_*b0*_*/f*_*c0*_: Ratio of equibiaxial to uniaxial compressive yield stress in CDP
*d*_*c*_: Compression damage parameter (0 = undamaged, 1 = fully damaged)
*d*_*t*_: Tension damage parameter (0 = undamaged, 1 = fully damaged)
*E*_*s*_: Modulus of elasticity of reinforcing steel
*u₁*, *u₂*, *u₃*: Displacement components in global directions for boundary conditions
*V*_*u*__*,EXP*_: Experimental ultimate shear load
*V*_*u*__*,NUM*_: Numerically predicted ultimate shear load
*∆*_*u*__*,EXP*_: Experimental mid-span ultimate deflection
*∆*_*u*__*,NUM*_: Numerical mid-span ultimate deflection
